# Innovative Approaches on the Estimation of the Effective Permittivity of Fibrous Media

**DOI:** 10.3390/ma18010014

**Published:** 2024-12-24

**Authors:** Jesus Nain Camacho Hernandez, Guido Link

**Affiliations:** Institute for Pulsed Power and Microwave Technology IHM, Karlsruhe Institute of Technology, Hermann-von-Helmholtz-Platz 1, 76344 Eggenstein-Leopoldshafen, Germany

**Keywords:** fibers, fabrics, effective permittivity, tensor permittivity

## Abstract

Estimating the effective permittivity of anisotropic fibrous media is critical for advancing electromagnetic applications, requiring detailed microstructural and orientation analyses. This study introduces innovative approaches for disclosing the orientation and microstructure of fibers, leading to mixing relations. It particularly focuses on two specific fiber configurations: 1. wave-curved fibers and 2. a collection of interconnected fibers. The first approach uses sinusoidal wave fibers, considering their curvature and direction. Conversely, the approach for the interconnected fibers operates on the principle of representing fibers as a collection of straight segments. Investigations on fibrous media for both approaches were performed using numerical calculations at the microwave frequency of 2.45 GHz. Each fibrous medium was treated as an effective medium by using fibers significantly smaller than the microwave wavelength. A thorough comparison was made between the proposed mixing relations, numerical data, and state-of-the-art mixing relations to assess their consistency and validity. The comparison of the proposed approaches with traditional models shows an improved accuracy of up to 70% and 8% for the real and imaginary components of the permittivity, respectively. Additionally, the root-mean-square errors were determined as 0.001 + *j*0.003 and 0.001 – *j*0.007 for the sinusoidal and interconnected straight fibers approaches, respectively. In addition, a woven alumina fabric was used to compare the experimental resonance frequency with that from simulations using the permittivity of the fabric estimated by the interconnected straight fibers approach. These findings advance the predictive accuracy of permittivity estimation in fibrous media, providing a robust foundation for engineering applications.

## 1. Introduction

The permittivity of fibers plays a crucial role in various electromagnetic applications involving woven and non-woven fabrics. This includes electromagnetic functional textiles [1], fiber-reinforced composites used in circuits [2], and fabric antennas [3]. The permittivity of these fibrous materials corresponds to their interaction with electromagnetic fields, thereby significantly affecting key properties like signal transmission, electromagnetic shielding effectiveness, and the performance of antennas.

A fibrous medium featuring fibers significantly smaller than the wavelength of propagating electromagnetic radiation can be treated (under the quasi-static assumption) as an effective medium. In consequence, the medium exhibits a characteristic effective permittivity $\varepsilon_{eff}$. This effective permittivity is influenced by diverse factors, such as the solid volume fraction of fibers and the permittivity of the fibers and host medium, as well as the orientation and geometry of the fiber microstructure.

Describing $\varepsilon_{eff}$ by using mixing relations [4,5] involves employing either probability distribution relations based on the micromechanical bounds or mixing relations based on the Effective Media Approximation (EMA relations) [4]. Probability distribution relations involve correlating $\varepsilon_{eff}$ with weighting parameters based on empirical data and micromechanical bounds. These micromechanical bounds, such as Hashin–Shtrikman or Wiener bounds [5], are used to establish limits on the possible values for $\varepsilon_{eff}$ in isotropic and anisotropic media, respectively. However, it is important to note that the accuracy of probability distribution relations depends significantly on the data acquisition process. This necessitates an experimental methodology that is capable of measuring the anisotropic $\varepsilon_{eff}$ of samples. Consequently, the fitting procedure should only account for the inclusion in which their orientations correspond to those in the measured samples. For other samples, a thorough characterization is required whenever their orientation varies.

EMA relations offer a practical alternative approach for estimating $\varepsilon_{eff}$. Unlike probability distribution relations that rely on empirical data, EMA relations utilize semi-analytical functions to estimate $\varepsilon_{eff}$. EMA relations assume that mixtures are composed of spherical or ellipsoidal inclusions embedded in a continuous medium, which are equally exposed to a volume-averaged electric field [6]. It is important to note that to maintain a valid mean electric field, significant attenuation of the field amplitude must be avoided. This condition is satisfied when the size of the inclusions is significantly smaller than the skin depth of the electromagnetic radiation.

The EMA framework estimates the macroscopic dielectric properties of materials by averaging the contributions of their individual inclusions. This framework relies on the principle that when a dielectric inclusion is placed in a uniform electric field E, it induces a secondary electric field within the inclusion. This induced field causes charges within the inclusion to align, generating a dipole moment p, which, in turn, alters the surrounding electric field. For moderate applied fields, the relationship between incident E and generated p is linear, given by p=α·E, where α is the polarizability of the inclusion. The Clausius-Mossotti formula [4] relates the macroscopic $\varepsilon_{eff}$ of a material to the combined contributions of the α of its microscopic inclusions. For one or more inclusion species, it is expressed as

(1) $$\frac{\varepsilon_{eff}-\varepsilon_{m}}{\varepsilon_{eff}+2\varepsilon_{m}}=\frac{1}{3\varepsilon_{m}}\sum_{i}n_{i}\alpha_{i},\;$$

where $\varepsilon_{m}$ is the permittivity of the continuous medium (host phase), and $n_{i}$ is the number density of dipoles of the *i*-th component (or species). In addition, the polarizability of a spherical inclusion is expressed as $\alpha_{i}=3V_{sph}\left(\varepsilon_{i}-\varepsilon_{m}\right)\varepsilon_{m}/\left(\varepsilon_{i}+2\varepsilon_{m}\right)$ [4], where $V_{sph}$ is the volume of the sphere, and $\varepsilon_{i}$ is the permittivity of the inclusion, respectively. The Clausius–Mossotti formula, using the polarizability of a spherical inclusion, is commonly referred to as the Maxwell–Garnett (MG) mixing relation [4]. The MG approach assumes a system with a low volume fraction of particles that are sufficiently spaced apart. This relation reads as

(2) $$\frac{\varepsilon_{eff}-\varepsilon_{m}}{\varepsilon_{eff}+2\varepsilon_{m}}=\sum_{i}\delta_{i}\frac{\varepsilon_{i}-\varepsilon_{m}}{\varepsilon_{i}+2\varepsilon_{m}},\;$$

where $\delta_{i}$ is volume fraction of the *i*-th component given by $\delta_{i}=n_{i}V_{sph}$.

Other frequently used EMA relations include the Bruggeman–Landauer (BL) mixing relation [4,5,6]. The BL relation is derived from the MG relation by assuming that $\varepsilon_{m}=\varepsilon_{eff}$ and treating the host medium as an inclusion phase, such that $\sum \delta_{i}=1$ [7]. The Bruggeman approach considers the interactions between induced dipoles of particles in a concentrated particle system. Unlike the MG relation, the BL relation symmetrically treats the volume fraction dispersion of the inclusions $\delta_{i}$ and host medium $\delta_{m}=1-\delta_{i}$. For a multiphase mixture, the BL relation is given as

(3) $$\left(1-\sum_{i}\delta_{i}\right)\frac{\varepsilon_{m}-\varepsilon_{eff}}{\varepsilon_{m}+2\varepsilon_{eff}}+\sum_{i}\delta_{i}\frac{\varepsilon_{i}-\varepsilon_{eff}}{\varepsilon_{i}+2\varepsilon_{eff}}=0\;.$$

Both MG and BL relations produce good estimates of permittivity in isotropic media. However, fibrous media characterized by a dominant fiber orientation exhibit anisotropic behavior, leading to the requirement of a second-rank tensor description for precise representation of their effective permittivity. Anisotropic EMA relations provide a means to estimate the permittivity tensor by incorporating ellipsoidal inclusions and considering their spatial orientation with respect to their ellipsoidal depolarization factors. Nevertheless, when dealing with microstructures that are geometrically not analogous to ellipsoids (i.e., curved fibers), the effectiveness of EMA relations in providing reliable estimates diminishes. In such cases, alternative approaches are typically preferred to accurately estimate the effective permittivity.

For geometries other than spheres or ellipsoids, an alternative approach involves using numerical calculations to determine the polarizability of the inclusions. After deriving a polarizability expression, it can be integrated into the Clausius–Mossotti formula to calculate $\varepsilon_{eff}$. While analytical expressions for the scattered fields can be derived for spherical and ellipsoidal inclusions, more complex inclusion shapes require numerical calculations to obtain polarizability expressions. These calculations are essential to identify higher-order functions that accurately represent the induced dipole moment. In such cases, the induced dipole moment is numerically approximated and expressed in relation to the volume and the permittivity of both the inclusions and the host media [8,9,10]. This approach is effective for estimating $\varepsilon_{eff}$ in cases where inclusions share the same geometry. However, its applicability is limited in most fibrous media, as the varying curvature of fibers results in significant geometric differences from one inclusion to another.

Recent advancements in effective media theory, such as the Bragg–Pippard (BP) theory and Strong Property Fluctuation Theory (SPFT), address the limitations of conventional models like MG and BL [11]. BP improves on MG with depolarization dyadics that match actual particle shapes. SPFT treats inclusions as constitutive property fluctuations, using volume integral equations and statistical measures to derive effective parameters. Despite these advancements, challenges persist in characterizing inclusions with complicated structures [11], highlighting the need for further innovation to address the diversity and intricacy of modern composite materials.

Lastly, another approach to estimate $\varepsilon_{eff}$, particularly for non-spheroidal inclusions, involves characterizing the inclusion’s geometric shape. One approach to describing these shapes is through the spectral density function method [12]. Another viable option is employing mixing relations that directly establish a correlation between $\varepsilon_{eff}$ and the specific topology of the inclusions [13,14]. An example of this approach is a topological relation that has shown good $\varepsilon_{eff}$ estimations for both isotropic (i.e., open-cell foams [13]) and anisotropic (straight filaments [14]) media. The corresponding topological mixing relation is

(4) $$\varepsilon_{eff}=\frac{-2\delta_{m}}{\left(1+{\delta_{m}}^{2}\right)}\left(\varepsilon_{i}-\varepsilon_{m}\right)+\left(\varepsilon_{i}+g\varepsilon_{m}\right),$$

where g is a scalar complex-valued correlation parameter containing the topological details of the microstructure of the inclusions. Specifically, g integrates the volume fraction, orientation, geometry, and permittivity contrast between the inclusions and the continuous medium. In the case of isotropic media, g can be represented as a single value, sufficiently covering all these parameters. In contrast, for anisotropic media, it is necessary to detail the orientation of the inclusions in addition to the other parameters, i.e., $g=f\left(\delta_{i},\delta_{m},\varepsilon_{i},\varepsilon_{m},T,K\right)$. Thus, specifying $g_{\Lambda }$ to the principal Λ-directions (Λ = xx, yy or zz) is achieved as

(5) $$g_{\Lambda }=\delta_{i}\delta_{m}\varepsilon_{i}{\varepsilon_{m}}^{-1}\sum_{a=1}^{3}\sum_{b=1}^{3}\left|t_{\Lambda ,ab}\right|\kappa_{ab}\;,$$

where $t_{\Lambda ,ab}$ and $\kappa_{ab}$ denote the following components: the orientation tensor $T_{\Lambda }$ after a rotation to the principal Λ-directions and the geometrical correlation parameter K, respectively. Note that $\left|t_{\Lambda ,ab}\right|$ refers to the absolute value of $t_{\Lambda ,ab}$. Accordingly, the inclusion’s geometry is represented as

(6) $$Κ=\left[\begin{array}{ccc} \kappa_{11} & \kappa_{12} & \kappa_{13}\\ \kappa_{21} & \kappa_{22} & \kappa_{23}\\ \kappa_{31} & \kappa_{32} & \kappa_{33} \end{array}\right]\;,$$

while their orientation is characterized by

(7) $$T_{\Lambda }=\left[\begin{array}{ccc} t_{\Lambda ,11} & t_{\Lambda ,12} & t_{\Lambda ,13}\\ t_{\Lambda ,21} & t_{\Lambda ,22} & t_{\Lambda ,23}\\ t_{\Lambda ,31} & t_{\Lambda ,32} & t_{\Lambda ,33} \end{array}\right]\;.$$

As implied previously, $T_{\Lambda }$ denotes the orientation tensor after it has undergone a rotation, with the subscript Λ indicating the direction of the rotation. The original (non-rotated) orientation tensor T, describing the average inclusion directions, is given by

(8) $$T=\left[\begin{array}{ccc} t_{11} & t_{12} & t_{13}\\ t_{21} & t_{22} & t_{23}\\ t_{31} & t_{32} & t_{33} \end{array}\right]\;.$$

The rotated orientation tensor $T_{\Lambda }$ is computed using matrix multiplication with the basic rotation matrices $R_{x}\left(\theta \right)$, $R_{y}\left(\varphi \right)$ and $R_{z}\left(\varphi \right)$. Specifically $T_{\Lambda }=R_{\Lambda }TR_{\Lambda }^{T}$, where $R_{\Lambda }$ is the combined rotation matrix $R_{\Lambda }\left(\theta ,\varphi ,\varphi \right)=R_{x}\left(\theta \right)R_{y}\left(\varphi \right)R_{z}\left(\varphi \right)$ expressed as

(9) $$R_{\Lambda }=\left[\begin{array}{ccc} cos\varphi \;cos\varphi & -cos\varphi \;sin\varphi & sin\varphi \\ cos\theta \;sin\varphi +cos\varphi \;sin\theta \;sin\varphi & cos\theta \;cos\varphi -sin\theta \;sin\varphi \;sin\varphi & -cos\varphi \sin\theta \\ sin\theta \;sin\varphi -cos\theta \;cos\varphi \;sin\varphi & cos\varphi \;sin\theta +cos\theta \;sin\varphi \;sin\varphi & cos\theta \;cos\varphi \end{array}\right]\;.$$

The re-orientation towards the Λ-directions is accomplished by specifying the following Euler angles: $R_{xx}$(θ=0°,φ=0°,φ=0°), $R_{yy}$(θ=0°,φ=0°,φ=90°), and $R_{zz}$(θ=0°,φ=90°,φ=0°). For reference, $T_{xx}$ corresponds to the initial orientation tensor T.

Consequently, for anisotropic media, where the permittivity Λ-components (diagonal components of the tensor) are required, the topological relation is formulated as

(10) $$\varepsilon_{eff,\Lambda }=\frac{-2\delta_{m}}{\left(1+{\delta_{m}}^{2}\right)}\left(\varepsilon_{i}-\varepsilon_{m}\right)+\varepsilon_{i}\left(1+\delta_{i}\delta_{m}\sum_{a=1}^{3}\sum_{b=1}^{3}\left|t_{\Lambda ,ab}\right|\kappa_{ab}\;\right).$$

This relation facilitates the redirection of the inclusions through tensor rotation of T, enabling the permittivity evaluation in any specified Λ-direction. Note that both T and K are symmetric, meaning $t_{ab}=t_{ba}$ and $\kappa_{ab}=\kappa_{ba}$.

Finally, in addition to mixing relations, the dielectric properties of materials with inclusions (e.g., fabrics [15,16]) can also be determined by using an electrical capacitance approach. This approach involves correlating the effective dielectric constant $\varepsilon_{eff}^{\prime }$ (real part of $\varepsilon_{eff}$) with the capacitance C observed in a parallel plate capacitor, that is

(11) $$\varepsilon_{eff}^{\prime }=\frac{L}{\varepsilon_{0}A}C,$$

where A and L are the parallel area and gap between parallel plates, respectively. The calculation of C involves describing the total length of fiber material between the parallel plates by considering the weave pattern of the interlaced fibers [15]. However, this approach is best suited for estimating the permittivity of materials with inclusions arranged in a well-defined structure, i.e., woven fibers with a repeating weave. Conversely, it is not suitable for randomly oriented fibers found in non-woven fabrics.

This work investigates the structural geometry and orientation of anisotropic fibrous media, introducing two novel approaches for estimating *ε*_eff_:

1. The first approach considers **sinusoidal wave-curved fibers**. Unlike conventional EMA models assuming ellipsoidal inclusions, the proposed wave-curved model captures complex fiber undulations, providing higher accuracy.
2. The second approach treats fibers as a collection of **interconnected straight elements**, overcoming the first approach’s limitation to strictly consider sinusoidal waves and enabling the approximation of any curvature without an explicit geometric characterization.

In addition, the present investigation focuses on assessing the accuracy of these approaches in estimating *ε*_eff_ for various fibrous media, as follows:

- Dispersed fibers: *ε*_eff_ estimates from the proposed approaches with the conventional EMA models and electromagnetic wave propagation simulations to assess accuracy for spread-out fibers.
- Plain-woven fabrics: the comparison for woven fabrics is similar to that for dispersed fibers, including the proposed approaches, EMA conventional mixing relations, and numerical calculations, with the addition of a theoretical capacitance model for woven constructions.
- Experimental woven fabrics: The resonance frequency of a woven alumina fabric in a microwave resonator is compared experimentally with that from numerical simulations, where the permittivity of the fabric is estimated using the proposed approaches applied for woven fabrics.

The manuscript’s structure is outlined as follows: Section 2 presents the methodologies used for estimating *ε*_eff_, including numerical calculations, conventional EMA models, the proposed novel approaches, and a revised capacitance model from the literature. This section also describes the experimental procedure and characteristics of a fibrous sample used for experimental confirmation. Section 3 compares the methods’ estimates, emphasizing which approach provides the best estimates for simulated dispersed fibers, simulated woven fabrics, and an experimental alumina fabric. Section 4 summarizes the key findings and limitations of the analyzed approaches.

## 2. Materials and Methods

### 2.1. Numerical Calculation of the Effective Permittivity

**Figure 1:**
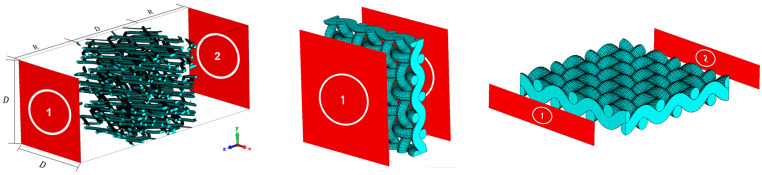
Examples of two fibrous model simulations: (**left**) dispersed fibers and (**center** and **right**) woven fabrics. Models are depicted with red-colored ports.

Fibrous models representing two distinct categories—dispersed fibers and woven fabrics—were generated utilizing GeoDict (version 2023, Math2Market, Kaiserslautern, Germany). These models were then imported into CST Studio (CST 2022, Dassault Systems, Velizy-Villacoublay, France) to conduct electromagnetic wave propagation simulations to determine their scattering parameters, as shown in Figure 1.

Following findings from a previous parametric analysis [14], the dispersed fibers models were incorporated into the simulations within cubes with a dimension $D=30d_{f}$, where $d_{f}$ is the fiber diameter. This specification, coupled with a voxel resolution of $d_{f}/10$, has been proven to ensure an optimal fiber resolution.

**Figure 2:**
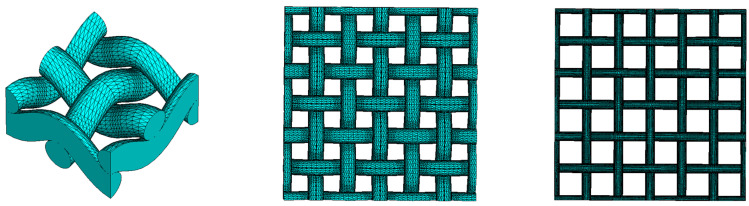
Representation of a woven fabric unit cell (**left**) and two woven fabric models (**center** and **right**) featuring different *δ*_m_ of 0.505 and 0.764, respectively.

Woven fabric models were simulated by using models conformed of three-unit cells (as shown in Figure 2, left), with both warp and weft yarns sharing the same $d_{f}$. Porosity (or $\delta_{m}$) was adjusted by modifying the spacing between successive warp and weft yarns while maintaining the same $d_{f}$ (see Figure 2, center and right).

The boundary conditions for both fibrous models were selected to generate an electromagnetic plane wave. This is achieved by implementing boundaries as normal electric walls $\left(E_{tangential}=0\right)$ and normal magnetic walls $\left(H_{tangential}=0\right)$ along the x- and *y*-axis, respectively. Furthermore, the *z*-axis boundaries are designed as non-reflective open boundaries at ports 1 and 2. An additional spatial consideration includes maintaining a distance of $R=25d_{f}$ from these ports to the disperse fibrous models, while for the fabric models, the distance is R=0.25 mm. This distance is set to minimize boundary effects, thereby ensuring accurate wave propagation with diminished boundary reflections and interference. For wave propagation parallel to the plane of the woven fabric model, the electric field is oriented perpendicular to the plane of the fabric. Using the frequency domain solver and an adaptive mesh refinement (with a convergence criterion of 0.02 for the absolute values of all S-parameters) in CST Studio, calculations were performed across a frequency range of 2.44 GHz to 2.46 GHz. Monitoring was specifically focused at 2.45 GHz to obtain the reflected $S_{11}$ and transmitted $S_{12}$ scattering signals. Monitoring was specifically focused at 2.45 GHz to obtain the reflected $S_{11}$ and transmitted $S_{12}$ scattering signals. This frequency was selected based on previous simulations [13,14], where the distance between the model and the ports was found to provide reliable performance. For other frequency ranges, further study may be needed to optimize the distance between the ports and the model. Additionally, any microwave frequency could be utilized, provided the medium meets the conditions of an effective medium. Subsequently, $\varepsilon_{eff}$ and $\mu_{eff}$ were calculated from these scattering parameters by the retrieval method [17]. Given that the models lack magnetic properties, the primary purpose of computing $\mu_{eff}$ was to verify if the calculations yield the characteristic of a non-magnetic sample, specifically $\mu_{eff}=1.0-j0.0\pm \left(0.1-j0.005\right).$ Numerical calculations were conducted using a fiber permittivity of $\varepsilon_{i}=10.0-j5.0$ and a host medium permittivity (air) of $\varepsilon_{h}=1.0-j0.0$. The chosen value of $\varepsilon_{i}$ ensures sufficient contrast for a clear evaluation of the effective permittivity. Permittivity values were not varied, as a previous study [13] showed that varying the dielectric contrast has no significant effect on the effective permittivity, provided the material remains an effective medium.

### 2.2. EMA Mixing Relations for Anisotropic Media

The EMA mixing relations (i.e., the MG or Bruggeman relations), when applied to anisotropic media, require accounting for the anisotropic orientation of their inclusions. This orientation is quantitatively assessed through the depolarization factors $N_{\Lambda }=N_{xx},\;N_{yy},$ and $N_{zz}$ [4]. Specifically, ellipsoids that are infinitely elongated (approximating long cylinders or straight fibers) along an electric field directed to the *x*-axis have depolarization factors of $N_{xx}=0$, $N_{yy}=N_{zz}=0.5$. The MG mixing relation, incorporating the depolarization factors, is utilized to determine the principal tensor Λ-components (Λ = *xx, yy,* or *zz*) of the effective permittivity. In the case of a system composed of two distinct constituents (medium and inclusions), the MG relation reads as

(12) $$\varepsilon_{eff,\Lambda }=\varepsilon_{m}+\delta_{i}\varepsilon_{m}\frac{\varepsilon_{i}-\varepsilon_{m}}{\varepsilon_{m}+\left(1-\delta_{i}\right)N_{\Lambda }\left(\varepsilon_{i}-\varepsilon_{m}\right)}.$$

In a similar way, the Traditional Anisotropic Bruggeman–Landauer (TAB) mixing relation incorporating the depolarization factors [18,19,20] is given as

(13) $$\delta_{m}\frac{\varepsilon_{m}-\varepsilon_{eff,\Lambda }}{\varepsilon_{eff,n}+N_{\Lambda }\left(\varepsilon_{m}-\varepsilon_{eff,\Lambda }\right)}+\delta_{i}\frac{\varepsilon_{i}-\varepsilon_{eff,\Lambda }}{\varepsilon_{eff,n}+N_{\Lambda }\left(\varepsilon_{i}-\varepsilon_{eff,\Lambda }\right)}=0.$$

### 2.3. Describing the Fibrous Microstructure by Using Wave-Curved Fibers

**Figure 3:**
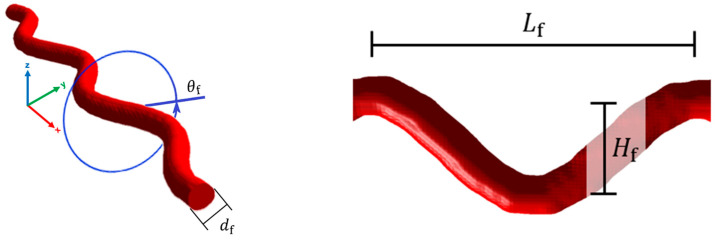
A sinusoidal wave-curved fiber is depicted, indicating (**left**) the fiber’s diameter and the angle of the curvature direction, as well as (**right**) the height and length of one of its curvature segments.

Topological mixing relations employ a parameter Κ to establish a correlation with the microstructure of the inclusions. To derive a Κ parameter for curved fibers, we propose an approach that considers fibers as sinusoidal crimped filaments. These filaments are defined by the diameter $d_{f}$, height $H_{f}$, and length $L_{f}$ of their curvature segments, as well as the angle $\theta_{f}$ of the curvature direction with respect to its perpendicular cross-sectional plane, as illustrated in Figure 3. Moreover, $\theta_{f}$ = 0° is defined for fibers where the sinusoidal curvature (or undulation) is parallel aligned with the electric field and increases to 90° as it rotates to become perpendicular or not aligned to the electric field.

Numerical electromagnetic wave propagation calculations were employed to determine $\varepsilon_{eff}$ for various models of specifically oriented sinusoidal wave-curved fibers. This method seeks to model the parameter Κ as a function of the fibers’ geometrical parameters, denoted as $Κ=f\left(d_{f},H_{f},L_{f},\theta_{f}\right)$. Detailed numerical analyses were performed to delineate the fibrous medium’s microstructure, employing the $\kappa_{ab}$ parameters (components of K) used in the topological mixing relation (Equation (10)). The wave-curved fiber models were created in GeoDict with a fiber diameter of $d_{f}=10\;\mu m$ and ensuring no fiber overlap. The selected diameter size falls within the micrometer range, where the microwave wavelength is significantly larger, thereby satisfying the conditions for the effective medium approximation. The investigation explored the wave-curved fibers through the next geometrical parameters, as follows:
I.$H_{f}{d_{f}}^{-1}=1,\;2,\;3,\;5,\;20,\;30$II.$L_{f}{d_{f}}^{-1}=11.25,\;16.875,\;22.5,\;40,\;60$III.$\theta_{f}=0{}^{\circ},\;30{}^{\circ},\;60{}^{\circ},\;and\;90{}^{\circ}$

**Figure 4:**
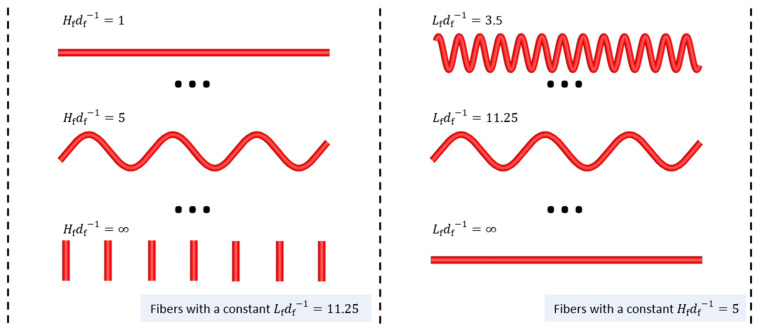
Schematic representation of wave-curved fibers, highlighting geometric parameters defining the curvature of the fibers.

**Figure 5:**
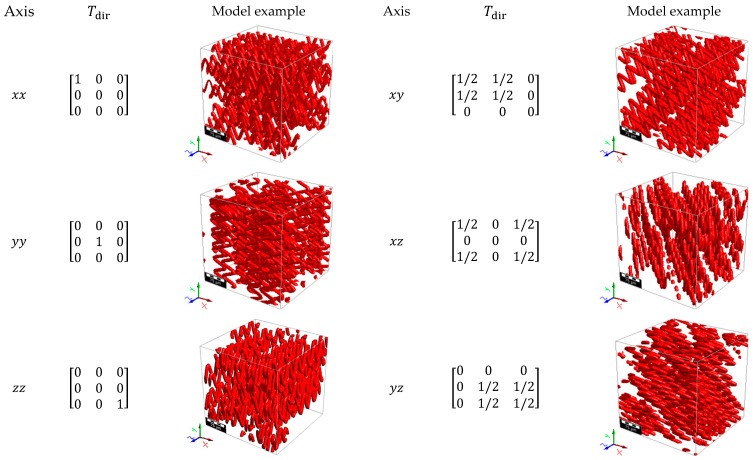
Illustration of fiber model examples directed to the Cartesian coordinate axes.

Figure 4 illustrates wave-curved fibers with varying $H_{f}{d_{f}}^{-1}$ and $L_{f}{d_{f}}^{-1}$ values, demonstrating their influence on fiber curvature. The direction of the fibers was defined through an orientation tensor $T_{dir}$ that describes the direction of the wave in relation to the Cartesian coordinate axes $\left\{x,xy,xz,y,yz,z\right\}$, as illustrated in Figure 5. Note that $T_{dir}$ represents the average fiber direction based on its endpoints within the domain, whereas T indicates the average spatial orientation of each fiber section, extending from an internal point to the structure’s boundary in all directions. Therefore, $T_{dir}$ indicates the overall direction in which the fibers are aligned, whereas T offers a more detailed insight into the fibers’ morphology.

**Figure 6:**
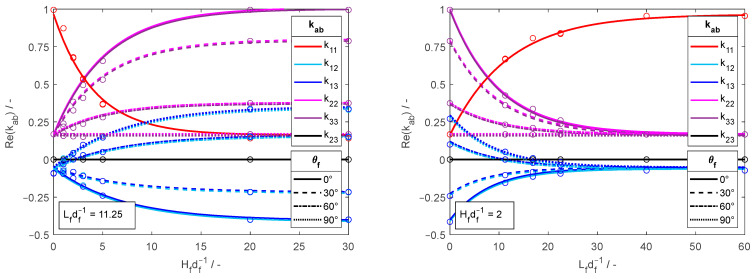

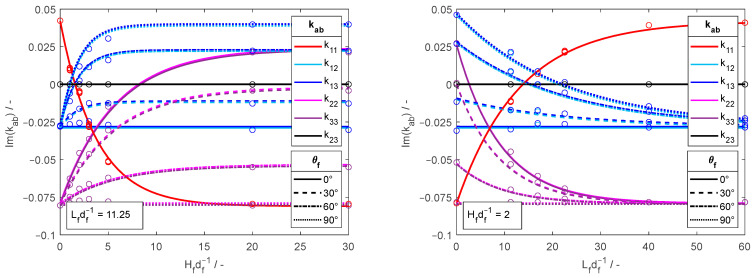
Variation of the real and imaginary components of $\kappa_{ab}$ with $H_{f}d_{f}^{-1}$ and $L_{f}d_{f}^{-1}$ for various $\theta_{f}$ values. The left graphs vary $H_{f}d_{f}^{-1}$ holding $L_{f}d_{f}^{-1}=11.25$, whereas the right ones vary $L_{f}d_{f}^{-1}$ maintaining $H_{f}d_{f}^{-1}=2$.

Examples of calculated $\kappa_{ab}$ parameters are presented in Figure 6. The data fit to exponential and linear trendlines (using $T_{dir,fN}$ as T), and a good approximated ($R^{2}>0.96$) was achieved by employing the next expression,

(14) $$\kappa_{ab}=\left[M_{ab}+J_{\left(\theta_{f},ab\right)}{\left(\frac{\left(C_{\left(\theta_{f},ab\right)}-M_{ab}\right)\cdot \left(1-e^{B_{ab}\cdot H_{f}d_{f}^{-1}}\right)}{J_{\left(\theta_{f},ab\right)}}\right)}^{\frac{L_{f}d_{f}^{-1}}{11.25}}\right]+j\left[P_{ab}+N_{\left(\theta_{f},ab\right)}{\left(\frac{\left(F_{\left(\theta_{f},ab\right)}-P_{ab}\right)\cdot \left({1-e}^{E_{ab}\cdot H_{f}d_{f}^{-1}}\right)}{N_{\left(\theta_{f},ab\right)}}\right)}^{\frac{L_{f}d_{f}^{-1}}{11.25}}\right]\;,$$

where $C_{\left(\theta_{f},ab\right)},F_{\left(\theta_{f},ab\right)},J_{\left(\theta_{f},ab\right)},N_{\left(\theta_{f},ab\right)},B_{ab},E_{ab},M_{ab},$ and $P_{ab}$ (refer to Table 1) correspond to the ab  components of symmetric matrices used for adjusting the amplitude, growth rate, and baseline of the trendlines.

**Figure 7:**
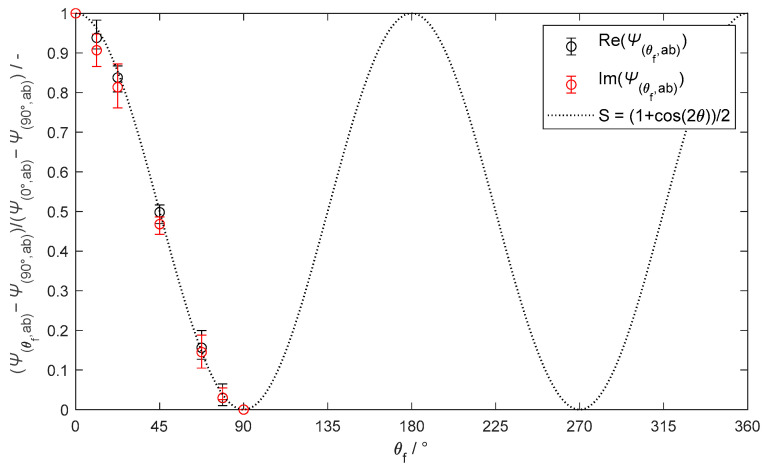
Variation of the normalized difference $\left(\Psi_{\left(\theta_{f},ab\right)}-\Psi_{\left(90{}^{\circ},ab\right)}\right)/\left(\Psi_{\left(0{}^{\circ},ab\right)}-\Psi_{\left(90{}^{\circ},ab\right)}\right)$ with $\theta_{f}$. The variation is approximated by the function S, which reproduces the symmetry every 90°.

The fibrous models exhibit a repetitive pattern in a function of $\theta_{f}$ with symmetries occurring at 90° intervals. The pattern has reflectional symmetry between 90° and 270° and rotational symmetry between 0° and 180° (for instance, $\theta_{f}=30{}^{\circ}$ is equal to $\theta_{f}=150{}^{\circ},210{}^{\circ},$ and 330°). The components $C_{\left(\theta_{f},ab\right)},\;F_{\left(\theta_{f},ab\right)},\;J_{\left(\theta_{f},ab\right)},\;and\;N_{\left(\theta_{f},ab\right)},$ which change with $\theta_{f},$ have been collectively denoted as $\Psi_{\left(\theta_{f},ab\right)}$. Figure 7 depicts the variation of $\Psi_{\left(\theta_{f},ab\right)}$ with $\theta_{f}$ via a normalized difference formula: $\left(\Psi_{\left(\theta_{f},ab\right)}-\Psi_{\left(90{}^{\circ},ab\right)}\right)/\left(\Psi_{\left(0{}^{\circ},ab\right)}-\Psi_{\left(90{}^{\circ},ab\right)}\right).$ This variation is well approximated $\left(R^{2}=0.998\right)$ by a function $S=\left(1+\cos\left(2\theta_{f}\right)\right)/2,$ enabling the evaluation of the $\Psi_{\left(\theta_{f},ab\right)}$ coefficients as

(15) $$\Psi_{\left(\theta_{f},ab\right)}=\left(\Psi_{\left(0{}^{\circ},ab\right)}-\Psi_{\left(90{}^{\circ},ab\right)}\right)S+\Psi_{\left(90{}^{\circ},ab\right)}.$$

### 2.4. Describing the Geometrical Microstructure by Using Interconnected Elements

**Figure 8:**
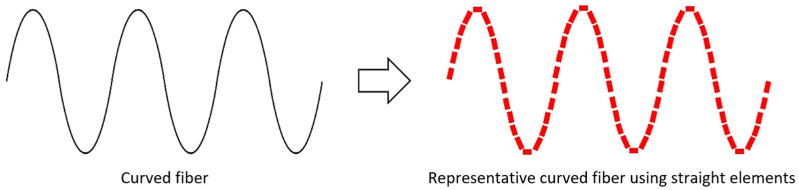
Structure (**left**) of a sinusoidal wave-curved fiber represented by (**right**) a collection of short straight filaments.

A second approach considers fibers as a collection of interconnected elements, such as straight filaments, to approximate curved fibers, as illustrated in Figure 8. Given that Κ is independent of the size of the straight filament, as long as it is significantly smaller than the electromagnetic wavelength, straight elements arranged to represent a curved fiber could provide a good approximation to estimate their effective permittivity. To ensure accurate representation, the orientation description of the straight filaments should match the same orientation tensor T as that of the curved fiber.

In contrast to a previous work [14], which focused on the disclosure of the $\kappa_{ab}$ components (of parameter Κ) for straight filaments aligned with principal axis directions, the present study introduces a wider range of porosity and fiber orientations to calculate $\kappa_{ab}$. This enhancement has resulted in a more precise determination of the $\kappa_{ab}$ components. In addition, the diagonal $\kappa_{ab}$ components were found to vary with porosity in a manner analogous to the variations of the Wiener $\varepsilon_{w}$ and Hashin–Shtrikman $\varepsilon_{HS}$ bounds. Specifically, $\kappa_{11}$ aligns with the upper Wiener bound $\varepsilon_{w}^{+}$, while $\kappa_{22}$ and $\kappa_{33}$ fall between the lower HS and Wiener bounds at $\varepsilon_{HS}^{-}-\varepsilon_{w}^{-}=0.8865-j0.0229$, leading to their evaluation by setting Equation (10) equal to 0.8865−j0.0229. The refined $\kappa_{ab}$ values are summarized in Table 2. Also, it is noteworthy to mention that for porosities exceeding 0.85, the diagonal $\kappa_{ab}$ components closely match values previously reported for filamentous media with higher porosity [14].

The versatility of Κ suggests that the interconnected elements approach could be generalized to accommodate any inclusion’s geometry. Similarly, the elongated ellipsoids from the MG and TAB mixing relations can be arranged to mimic the geometry of fibers, offering a practical method for estimating their effective permittivity.

### 2.5. Describing Electrical Capacitance of Fabrics Based on Fiber Construction

**Figure 9:**
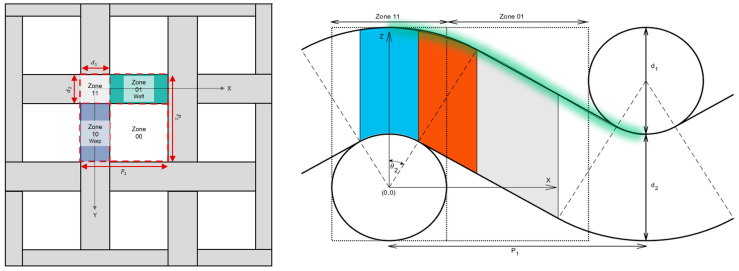
Plain-woven fabric (**left**) and its structure on the XZ plane (**right**), with its curvature highlighted in green.

Within a series capacitor, the capacitance of an elementary region containing a dielectric material between two parallel plates is expressed [16] as

(16) $$C=\varepsilon_{0}\int_{y_{1}}^{y_{2}}\int_{x_{1}}^{x_{2}}\frac{dxdy}{\frac{L-u}{\varepsilon_{m}^{\prime }}+\frac{u}{\varepsilon_{i}^{\prime }}}=\frac{\varepsilon_{0}}{L}\int_{y_{1}}^{y_{2}}\int_{x_{1}}^{x_{2}}\frac{dxdy}{\frac{1}{\varepsilon_{m}^{\prime }}-\left(\frac{1}{\varepsilon_{m}^{\prime }}-\frac{1}{\varepsilon_{i}^{\prime }}\right)\frac{u}{L}}\;,$$

where $\varepsilon_{0}$ is the permittivity of free space ($8.854\times 10^{-12}\;F\cdot m^{-1}$), u are the length of the fiber (or yarn) material along the *z*-axis, and L is the distance between the two parallel capacitor plates. The area limited by the differentials dx and dy corresponds to the plane parallel to the capacitor plates. The technique of deriving capacitance is based on defining the internal geometry of the fibers through the quantity of material u that lies in direction to the plates. Bal and Kothari [16] developed a structural model that represents the capacitance of a plain-woven fabric embedded in air ($\varepsilon_{m}^{\prime }\sim 1$). Their model considers weft and warp yarns (along the *y*- and *x*-axis, respectively) of diameter $d_{2}$ and $d_{1}$, respectively. These yarns form a woven fabric described by Peirce’s basic fabric geometry [20], which is represented in Figure 9.

Given Peirce’s configuration, the distances between successive warp and weft yarns are defined by $P_{1}\;and\;P_{2}$, respectively. These distances are inversely related to the thread counts of the warp and weft yarns as $n_{wp}=1/P_{1}\;and\;n_{wt}={1/P}_{2}$. Additionally, the length of warp and weft yarns is distinguished as $u_{wt}$ and $u_{wp}$, respectively. The angles of the weave for warp and weft yarns are denoted by $\theta_{1}$ and $\theta_{2}$.

Notably, the model as proposed by Bal and Kothari [18]) is only valid under specific conditions, as follows: $\left(d_{1}/2+d_{2}\right)sin\left(\theta_{2}\right)\leq d_{1}/2$ and $\left(d_{2}/2+d_{1}\right)sin\left(\theta_{1}\right)\leq d_{2}/2$. Thus, it does not apply to all possible weave angles of the yarns in a Peirce’s configuration. Therefore, the model has been revised to refine its applicability to those conditions that preserve a Peirce’s configuration. Following the methodology of Bal and Kothari, we categorize $u_{wt}$ and $u_{wp}$ into three distinct regions, as highlighted in Figure 9 (right) in blue (B), orange (O), and gray (G). Particular attention was given to the gray region, from which $u_{wt}$ and $u_{wp}$ (for $\left|y\right|\leq d_{2}/2$ and $\left|x\right|\leq d_{1}/2$, respectively) have been modified as

(17) $$u_{wt}\left(x,y\right)=\left\{\begin{array}{c} \left(\sqrt{{\left(\frac{d_{1}}{2}+d_{2}\right)}^{2}-x^{2}}-\sqrt{{\left(\frac{d_{1}}{2}\right)}^{2}-x^{2}}\right)\sqrt{1-{\left(\frac{2y}{d_{2}}\right)}^{2}}\;\;\;\;\;\;\;\;\;\;\;\;\;\;\;\;\;\;\;\;\;\;\;\;\;\;\;\;\;\;\;\;\;\;\;\;\;\;\;\;\;\;\;\;\;\;\;\;\;\;for\;\left|x\right|\leq \frac{d_{1}}{2}\;sin{\;\theta }_{2}\;\left(B\right)\\ \left(\sqrt{{\left(\frac{d_{1}}{2}+d_{2}\right)}^{2}-x^{2}}+x\tan\theta_{2}-\frac{d_{1}}{2}\sec\theta_{2}\right)\;\sqrt{1-{\left(\frac{2y}{d_{2}}\right)}^{2}}\;\;\;\;\;\;for\;\frac{d_{1}}{2}\;\sin\theta_{2}\leq \left|x\right|\leq \left(\frac{d_{1}}{2}+d_{2}\right)\sin\theta_{2}(O)\\ \left(d_{2}\cdot \sec\theta_{2}\right)\;\sqrt{1-{\left(\frac{2y}{d_{2}}\right)}^{2}}\;\;\;\;\;\;\;\;\;\;\;\;\;\;\;\;\;\;\;\;\;\;\;\;\;\;\;\;\;\;\;\;\;\;\;\;\;\;for\;\left(\frac{d_{1}}{2}+d_{2}\right)\sin\theta_{2}\leq \left|x\right|\leq P_{1}-\left(\frac{d_{1}}{2}+d_{2}\right)\sin\theta_{2}(G) \end{array}\right.,$$

(18) $$u_{wp}\left(x,y\right)=\left\{\begin{array}{c} \left(\sqrt{{\left(\frac{d_{2}}{2}+d_{1}\right)}^{2}-y^{2}}-\sqrt{{\left(\frac{d_{2}}{2}\right)}^{2}-y^{2}}\right)\sqrt{1-{\left(\frac{2x}{d_{1}}\right)}^{2}}\;\;\;\;\;\;\;\;\;\;\;\;\;\;\;\;\;\;\;\;\;\;\;\;\;\;\;\;\;\;\;\;\;\;\;\;\;\;\;\;\;\;\;\;\;\;\;\;\;for\;\left|y\right|\leq \frac{d_{2}}{2}\;\sin\theta_{1}(B)\\ \left(\sqrt{{\left(\frac{d_{2}}{2}+d_{1}\right)}^{2}-y^{2}}+y\tan\theta_{1}-\frac{d_{2}}{2}\sec\theta_{1}\right)\;\sqrt{1-{\left(\frac{2x}{d_{1}}\right)}^{2}}\;\;\;\;\;\;for\;\frac{d_{2}}{2}\;\sin\theta_{1}\leq \left|y\right|\leq \left(\frac{d_{2}}{2}+d_{1}\right)\sin\theta_{1}(O)\\ \left(d_{1}\cdot \sec\theta_{1}\right)\sqrt{1-{\left(\frac{2x}{d_{1}}\right)}^{2}}\;\;\;\;\;\;\;\;\;\;\;\;\;\;\;\;\;\;\;\;\;\;\;\;\;\;\;\;\;\;\;\;\;\;\;\;\;\;\;for\;\left(\frac{d_{2}}{2}+d_{1}\right)\sin\theta_{1}\leq \left|y\right|\leq P_{2}-\left(\frac{d_{2}}{2}+d_{1}\right)\sin\theta_{1}(G) \end{array}\;\right..$$

Note that Bal and Kothari advocated for calculating $\theta_{1}$ and $\theta_{2}$ by using a relationship that incorporates the weft and warp crimp values. However, for some waves, this may lead to breaching the fundamental Peirce’s geometrical model. In contrast, our analysis of the geometry reveals that $\theta_{1}$ and $\theta_{2}$ should be derived from the trigonometric characteristics inherent in Peirce’s configuration given by

(19) $$\left(P_{1}-\left(d_{1}+d_{2}\right)\cdot sin{\;\theta }_{2}\right)\cdot tan{\;\theta }_{2}+d_{2}-\left(d_{1}+d_{2}\right)\cdot cos{\;\theta }_{2}=0,$$

(20) $$\left(P_{2}-\left(d_{1}+d_{2}\right)\cdot sin{\;\theta }_{1}\right)\cdot tan{\;\theta }_{1}+d_{1}-\left(d_{1}+d_{2}\right)\cdot cos{\;\theta }_{1}=0.$$

By incorporating this modification, the model’s applicability is corrected but limited to weave angles that maintain a Peirce’s configuration. That is, as long as $P_{1}\geq \left(d_{1}+2d_{2}\right)sin\left(\theta_{2}\right)$ and $P_{2}\geq \left(d_{2}+2d_{1}\right)sin\left(\theta_{1}\right).$

The capacitance of the zones 11, 10, 01, and 00 are given as

(21) $$C_{Zone\;11}=\frac{\varepsilon_{0}}{L}\int_{-\frac{d_{2}}{2}}^{+\frac{d_{2}}{2}}\int_{-\frac{d_{1}}{2}}^{+\frac{d_{1}}{2}}\frac{dxdy}{\frac{1}{\varepsilon_{m}^{\prime }}-\left(\frac{1}{\varepsilon_{m}^{\prime }}-\frac{1}{\varepsilon_{i}^{\prime }}\right)\frac{u_{wt}\left(x,y\right)+u_{wp}\left(x,y\right)}{L}}\;,$$

(22) $$C_{Zone\;01}=\frac{\varepsilon_{0}}{L}\int_{-\frac{d_{2}}{2}}^{+\frac{d_{2}}{2}}\int_{+\frac{d_{1}}{2}}^{+\frac{P_{1}}{2}}\frac{2\;dxdy}{\frac{1}{\varepsilon_{m}^{\prime }}-\left(\frac{1}{\varepsilon_{m}^{\prime }}-\frac{1}{\varepsilon_{i}^{\prime }}\right)\frac{u_{wt}\left(x,y\right)}{L}},$$

(23) $$C_{Zone\;10}=\frac{\varepsilon_{0}}{L}\int_{+\frac{d_{2}}{2}}^{+\frac{P_{2}}{2}}\int_{-\frac{d_{1}}{2}}^{+\frac{d_{1}}{2}}\frac{2\;dxdy}{\frac{1}{\varepsilon_{m}^{\prime }}-\left(\frac{1}{\varepsilon_{m}^{\prime }}-\frac{1}{\varepsilon_{i}^{\prime }}\right)\frac{u_{wp}\left(x,y\right)}{L}},$$

(24) $$C_{Zone\;00}=\frac{\varepsilon_{0}}{L}\int_{+\frac{d_{2}}{2}}^{\left(P_{2}-\frac{d_{2}}{2}\right)}\int_{+\frac{d_{1}}{2}}^{\left(P_{1}-\frac{d_{1}}{2}\right)}\varepsilon_{m}^{\prime }\;dxdy=\frac{\varepsilon_{0}}{L}\varepsilon_{m}^{\prime }\left(P_{1}-d_{1}\right)\left(P_{2}-d_{2}\right).$$

The total capacitance is given as the cumulative sum of the capacitance (per unit area) from all zones scaled by the area of the parallel plates. This is expressed as

(25) $$C=\frac{\varepsilon_{0}An_{wp}n_{wt}}{L}\left(\int_{-\frac{d_{2}}{2}}^{+\frac{d_{2}}{2}}\int_{-\frac{d_{1}}{2}}^{+\frac{d_{1}}{2}}\frac{dxdy}{\frac{1}{\varepsilon_{m}^{\prime }}-\left(\frac{1}{\varepsilon_{m}^{\prime }}-\frac{1}{\varepsilon_{i}^{\prime }}\right)\frac{u_{wt}\left(x,y\right)+u_{wp}\left(x,y\right)}{L}}+\int_{-\frac{d_{2}}{2}}^{+\frac{d_{2}}{2}}\int_{+\frac{d_{1}}{2}}^{+\frac{P_{1}}{2}}\frac{2\;dxdy}{\frac{1}{\varepsilon_{m}^{\prime }}-\left(\frac{1}{\varepsilon_{m}^{\prime }}-\frac{1}{\varepsilon_{i}^{\prime }}\right)\frac{u_{wt}\left(x,y\right)}{L}}+\int_{+\frac{d_{2}}{2}}^{+\frac{P_{2}}{2}}\int_{-\frac{d_{1}}{2}}^{+\frac{d_{1}}{2}}\frac{2\;dxdy}{\frac{1}{\varepsilon_{m}^{\prime }}-\left(\frac{1}{\varepsilon_{m}^{\prime }}-\frac{1}{\varepsilon_{i}^{\prime }}\right)\frac{u_{wp}\left(x,y\right)}{L}}+\varepsilon_{m}^{\prime }\left(P_{1}-d_{1}\right)\left(P_{2}-d_{2}\right)\right)\;.$$

Finally, C is used in Equation (11) to calculate $\varepsilon_{eff}$. It is important to note that this extended capacitance model formulation does not set $\varepsilon_{m}^{\prime }=1$ (value for air or vacuum), allowing for considering fabrics embedded in any medium.

### 2.6. Experimental Measurements and Numerical Calculations Using a Split Resonator

**Figure 10:**
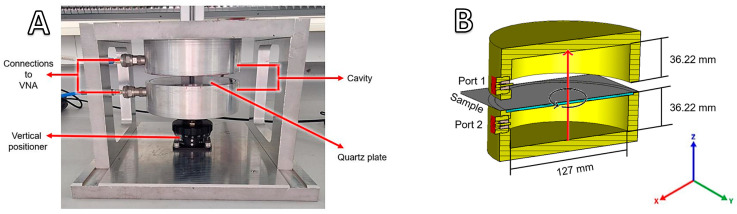
(**A**) Schematic of the experimental setup using a split cylindrical resonator and (**B**) its corresponding model for the numerical simulations.

The cavity perturbation method was chosen to assess the reliability of the $\varepsilon_{eff}$ estimates of an alumina fabric from mixing relations and experimental data. The setup used for experiments is based on a split cylinder resonator that is designed to excite the TE_111_ mode within the 2.45 GHz band. The TE_111_ mode has a linear polarized electric field in the equatorial plane (orthogonal to the *z*-axis) of the cavity, which allows for investigating the anisotropic permittivity of planar dielectric materials. Figure 10A shows the setup, and Figure 10B shows its dimensions. The setup includes a quartz plate supporter to hold samples and a micrometer vertical positioner, which allows for precise adjustment of the split distance. The resonator is characterized by a high Q-factor of around 10,417 when closed and 9984 at a split distance of 1 mm. Measurements of the scattering parameters and frequency were performed while rotating the fabric around the equatorial plane.

**Figure 11:**
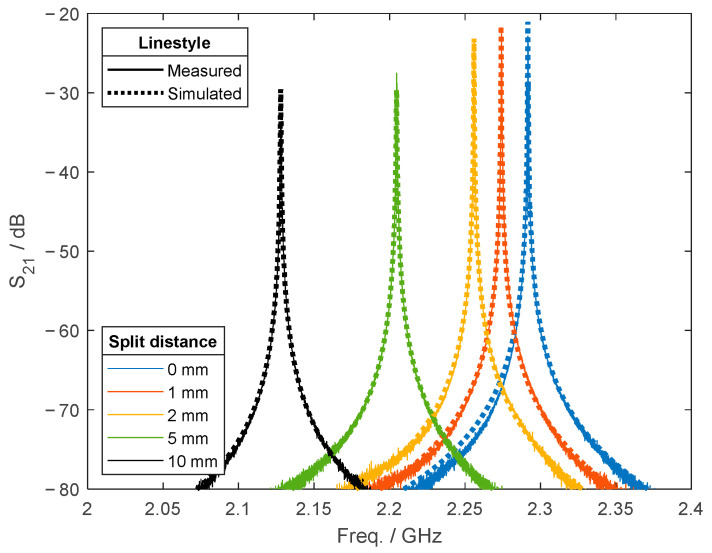
Comparison of the *S*_21_ parameter between the simulated and empty resonator at different split distances.

The measured scattering parameters were compared to those obtained from simulations of the split cylindrical model using CST Studio and are depicted in Figure 10B. Calculations were performed by defining the $\varepsilon_{eff}$ tensor of the fabric from the mixing relation based on interconnected straight fibers. In addition, $\varepsilon_{eff}$ was adjusted through tensor rotation, mirroring the experimental rotation of the fabric. Numerical calculations were conducted on the split resonator in the absence of a sample to validate the model’s alignment with the experimental setup. Figure 11 shows the $S_{21}$ parameter of the measured and simulated resonator at split distances of 0 (closed), 1, 2, 5, and 10 mm, demonstrating a good agreement of the resonance frequencies $f_{res}$.

### 2.7. Materials

**Figure 12:**
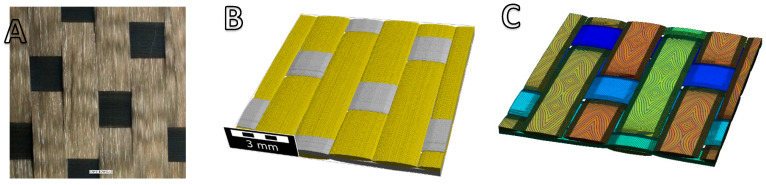
Microscopy image (**A**) showing a 20× magnified view of the DF-13-4500 fabric, (**B**) digital microstructure model (generated using Geo-Dict), and (**C**) voxel-based and segmented model.

The sample used in the split resonator measurements corresponds to a nearly pure alumina (Al_2_O_3_) known as Nextel™ 610 fiber fabric (a product of 3M Company). The fabric is identified by 3M as DF-13-4500 and is crafted in a 5-Harness-Satin weave pattern. Figure 12A depicts the fabric at a magnification of 20× with appropriate right illumination, captured using a digital microscope (model VHX-7000, Keyence GmbH, Neu-Isenburg, Germany). In Figure 12A, the machine and cross-direction yarns are clearly distinguishable, appearing in dark and light contrast, respectively. Table 3 presents a summary of the DF-13-4500 fabric properties.

A digital microstructure model of fabric DF-13-4500 was constructed by using Geo-Dict, as presented in Figure 12B. This model was subsequently voxel-based constructed and segmented (Figure 12C) in Matlab (R2022a, MathWorks Inc., Natick, MA, USA) for further analysis of its orientation tensor, which was analyzed by an in-house Star Length Distribution (SLD) algorithm [22].

## 3. Results and Discussion

### 3.1. Effective Permittivity in Fibrous Media: Numerical Calculations Compared to Mixing Relations

Numerical electromagnetic calculations were conducted to assess the accuracy of the wave-curved and the interconnected elements approaches (involving straight filaments and elongated ellipsoids from the MG and TAB), in estimating $\varepsilon_{eff,xx}$ for 12 specific fibrous models. The evaluated fibrous models include straight, sinusoidal, and multiple fibers; each with a fiber diameter of $d_{f}=10\;\mu m$.

**Figure 13:**
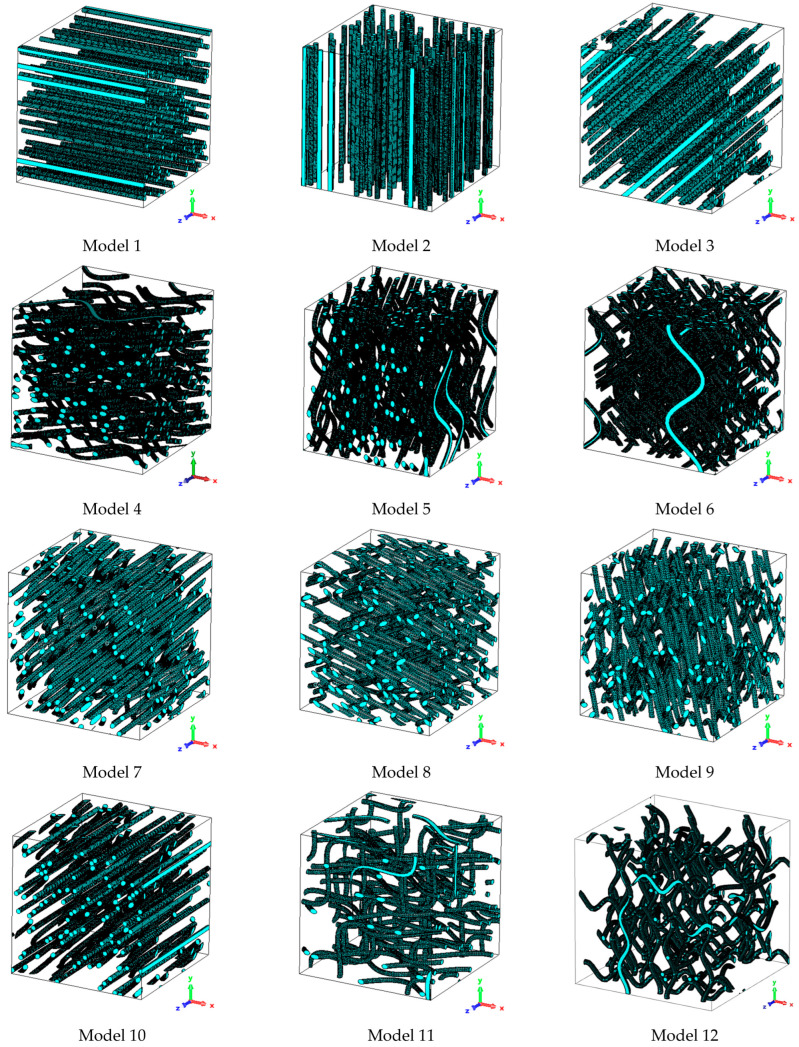
Visual representation of the evaluated fibrous models, categorizing them as: straight fibers (models 1–3), sinusoidal fibers (models 4–9), and composite fibers with multiple curvatures and orientations (models 10–12).

Models were organized into groups based on fibers sharing the same $T_{dir}$, forming a collective fN. Thus, models with multiple fibers feature several fN groups. Figure 13 offers a visual representation, while Table 4 details the properties of these models. The results of this comparison are presented in Table 5, with calculation examples of the mixing relations in Appendix A.1. These models, selected for their simple to complex geometry, offer a rigorous test for the approach’s estimate capability. The choice of these models is not intended to directly replicate real-world fibers geometries, but rather to provide a robust validation platform.

The decision to report *ε*_eff,xx_ was aimed at facilitating a straightforward comparison of a single and representative component direction rather than the full $\varepsilon_{eff}$ tensor, allowing for a more focused analysis of the models’ characteristics. Given that any diagonal component of $\varepsilon_{eff}$ is interchangeable through spatial rotation, i.e., $\varepsilon_{eff,xx}$ from models 2 and 5 becomes equivalent to $\varepsilon_{eff,yy}$ upon a 90° rotation around the *z*-axis, matching $\varepsilon_{eff,xx}$ from models 1 and 4.

The proposed approaches improved accuracy up to 70% and 8% for the effective dielectric constant and effective loss constant, respectively, based on the mean absolute error and compared with the Bruggeman relation, which outperforms the Maxwell–Garnett relation. Moreover, Table 1’s RMSE reveals that approaches utilizing sinusoidal wave-curved fibers yield the most accurate predictions across all evaluated approaches. This outcome aligns with expectations, given that the simulated models are sinusoidal fibers that are precisely characterized by the $\kappa_{ab}$ parameters from the sinusoidal wave-curved relation. The good agreement is explained, as the $\kappa_{ab}$ components effectively correlate the spatial contributions of the fibers’ curvature geometrical parameters (refer to Figure 6) with the components of the effective permittivity tensor due to localized distortions in the electric field.

Note that despite the results presented here, which consider only fibers with specific sinusoidal curvatures, the strength of the interconnected straight filament’s segments approach lies in their ability to approximate any curvature and is not limited to specific patterns.

### 3.2. Results of the Estimation of the Effective Permittivity in Plain-Woven Fabrics

This section focuses on comparing the previous mixing relations and the electrical capacitance description (from Section 2.4 and Section 2.5, respectively) against numerical calculations for estimating $\varepsilon_{eff}$ of woven fibers, particularly for plain-woven models. In plain-woven fabrics, the off-diagonal elements of the orientation tensor are zero, as the alternating up and down movement of yarns produces opposing off-diagonal components that cancel out. Conversely, the diagonal elements are related to the curvature of the yarns. The curvature is discernible from the top cross-section of their yarns, as shown in Figure 9 (right), and can be quantified through the gradient of their weft $m_{wt}$ and warp $m_{wp}$ yarns as

(26) $$m_{wt}=\left\{\begin{array}{c} \frac{-x}{\sqrt{{\left(\frac{d_{1}}{2}+d_{2}\right)}^{2}-x^{2}}}\;\;\;\;\;\;\;\;\;\;\;\;\;\;\;\;\;\;\;\;\;\;\;\;\;\;\;\;\;\;\;\;\;\;\;\;\;\;\;\;for\;\left|x\right|\leq \;\left(\frac{d_{1}}{2}+d_{2}\right)sin{\;\theta }_{2}\\ \frac{\left(d_{1}+d_{2}\right)\left(\frac{1}{2}-cos\left(\theta_{2}\right)\right)}{P_{1}-\left(d_{1}+d_{2}\right)sin\left(\theta_{2}\right)}\;\;\;\;\;\;\;\;\;\;\;\;\;\;\;\;\;\;\;\;\;\;\;\;\;\;\;\;\;\;\;\;\;\;\;\;\;for\;\left|x\right|\leq P_{1}-\frac{d_{2}}{2}sin{\;\theta }_{2}\\ \frac{\left(x-P_{1}\right)}{\sqrt{{\left(\frac{d_{1}}{2}\right)}^{2}-{\left(d_{2}-x\right)}^{2}}}\;\;\;\;\;\;\;\;\;\;\;\;\;\;\;\;\;\;\;\;\;\;\;\;\;\;\;\;\;\;\;\;\;\;\;\;\;\;\;\;\;\;\;\;\;\;\;\;\;\;\;\;\;\;\;\;\;\;\;\;\;\;\;for\;\left|x\right|\leq \;P_{1} \end{array}\right.\;,$$

(27) $$m_{wp}=\left\{\begin{array}{c} \frac{-y}{\sqrt{{\left(\frac{d_{2}}{2}+d_{1}\right)}^{2}-y^{2}}}\;\;\;\;\;\;\;\;\;\;\;\;\;\;\;\;\;\;\;\;\;\;\;\;\;\;\;\;\;\;\;\;\;\;\;\;\;\;\;\;for\;\left|y\right|\leq \;\left(\frac{d_{2}}{2}+d_{1}\right)sin{\;\theta }_{1}\\ \frac{\left(d_{2}+d_{1}\right)\left(\frac{1}{2}-cos\left(\theta_{1}\right)\right)}{P_{2}-\left(d_{2}+d_{1}\right)sin\left(\theta_{1}\right)}\;\;\;\;\;\;\;\;\;\;\;\;\;\;\;\;\;\;\;\;\;\;\;\;\;\;\;\;\;\;\;\;\;\;\;\;\;for\;\left|y\right|\leq P_{2}-\frac{d_{1}}{2}sin{\;\theta }_{1}\\ \frac{\left(y-P_{2}\right)}{\sqrt{{\left(\frac{d_{2}}{2}\right)}^{2}-{\left(d_{1}-y\right)}^{2}}}\;\;\;\;\;\;\;\;\;\;\;\;\;\;\;\;\;\;\;\;\;\;\;\;\;\;\;\;\;\;\;\;\;\;\;\;\;\;\;\;\;\;\;\;\;\;\;\;\;\;\;\;\;\;\;\;\;\;\;\;\;\;\;for\;\left|y\right|\leq \;P_{2} \end{array}\;\right.\;.$$

The diagonal elements are determined by the projection of the absolute and normalized means of the gradients of $m_{wt}$ and $m_{wp}$ by 90° onto the axial directions across the length of the plain-woven unit cell. Therefore, the orientation tensor for plain-woven fabrics, $T_{plain},$ is approximated by the sum of n-number of points equally distributed along the yarns’ curvature, as

(28) $$T_{plain}=\left[\begin{array}{ccc} \frac{1}{2n}\sum_{p=1}^{n}1-\frac{\left|\tan^{-1}\left(m_{wt,p}\right)\right|}{90{}^{\circ}} & 0 & 0\\ 0 & \;\;\;\frac{1}{2n}\sum_{p=1}^{n}1-\frac{\left|\tan^{-1}\left(m_{wp,p}\right)\right|}{90{}^{\circ}} & 0\\ 0 & 0 & \frac{1}{2n}\sum_{p=1}^{n}\frac{\left|\tan^{-1}\left(m_{wt,p}\right)\right|+\left|\tan^{-1}\left(m_{wp,p}\right)\right|}{90{}^{\circ}} \end{array}\right].$$

The models selected for numerical calculations are plain-weave fabrics utilizing weft and warp yarns with d=29 μm and feature a uniform distance P between successive warp and weft yarns ($P_{1}=P_{2}$). Properties including P, $T_{plain},$ and $\delta_{m}$ are provided in Table 6.

For applying the sinusoidal approach, both the weft and warp yarns are considered as sinusoidal fibers. The fiber’s curvature parameters $H_{f}$ and $L_{f}$ correspond to the yarn diameter d and to twice the yarn spacing 2P, respectively. As noted in Section 2.1, when the microwave propagates parallel to the fabric plane, the electric field is perpendicular to both weft and warp sinusoidal fibers. In this the case, the model can be simplified to a single fiber group (fN=1), with $\theta_{f}=90{}^{\circ}$, $\delta_{i}$ = $1-\delta_{m}$, and $T_{dir}$ corresponding to sinusoidal fibers aligned with either the y- or *z*-axis, that is, $\left[t_{11},\;t_{22},\;t_{33}\right]$ is equal to [0, 1, 0] or [0, 0, 1]. However, when the microwave propagates perpendicular to the fabric plane, the electric field direction can be parallel to the weft yarns and perpendicular to the warp yarns or vice versa. In this scenario, the sinusoidal approach must account for two fiber groups (fN=2), with the weft and warp fibers treated separately. Both fiber groups have $\theta_{f,fN}=0{}^{\circ}$ and $\delta_{i,fN}=(1-\delta_{m})/2$, but $T_{dir,fN}$ differs between them; for the $T_{dir,1},$ the diagonal elements $\left[t_{11},\;t_{22},\;t_{33}\right]=[1,\;0,\;0]$, while for $T_{dir,2},$ it is [0, 1, 0].

**Figure 14:**
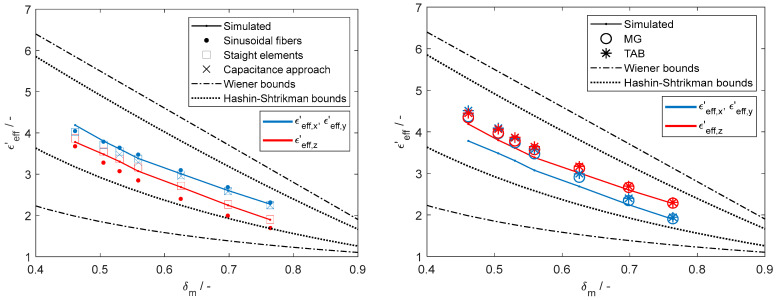
Comparison of the effective permittivity with porosity of the woven fabric models from numerical calculations estimated using interconnected straight elements, sinusoidal fibers, and the capacitance approach (**left**) and with ellipsoids (**right**) from the Maxwell–Garnett and Traditional Anisotropic Bruggeman mixing relations.

Figure 14 presents the estimated $\varepsilon_{eff}$ results, revealing that both the capacitance approach and the interconnected straight filaments model produce the best estimates. Conversely, the use of sinusoidal fibers and elongated ellipsoids from the MG and TAB models only provides satisfactory estimates as the porosity increases. For the sinusoidal approach, this is likely due to the contact between weft and warp yarns deviating from the idealized sinusoidal pattern. Furthermore, both the MG and TAB models rely on ellipsoidal elements, which are insufficient to represent the curvature of yarns. On the other hand, although the capacitance approach offers good estimates, its application is limited to a Peirce’s configuration, which for the analyzed fabric remains intact only for porosities above 0.5175. Consequently, the interconnected straight filaments approach, being unrestricted by a specific yarn configuration, stands out for its versatility in representing curved fibers (or yarns) while providing good agreement of $\varepsilon_{eff}$ estimates across a wider range of porosities.

### 3.3. Resonance Frequency and Permittivity of an Alumina Woven Fabric from Experimental Observations and the Interconnected Straight Filaments Approach

A plain-weave fabric featuring varying fibers in its weft and warp yarns generates an anisotropy tensor with distinct diagonal components from each other. In this study, an alumina fabric (discussed in Section 2.7) was used to tailor a sample with an anisotropic orientation tensor. This effect was achieved by removing four to seven fibers from each weft yarn, thus modifying both the orientation and effective permittivity tensors. Furthermore, the sample underwent a drying process for 12 h at 80 °C to eliminate water content. Measurements were conducted promptly after drying to minimize water reabsorption from ambient humidity. Table 7 provides a summary of the modified dried fabric sample, which was characterized by analyzing the yarns’ profile heights through digital analysis using a digital microscope as described in Section 2.

**Figure 15:**
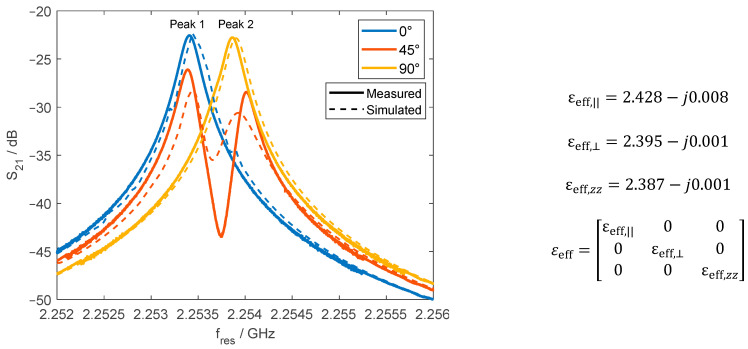
Resonance frequency shift (**left**) for each in-plane rotation of the sample and the corresponding calculated permittivity tensor (**right**) at 0° rotation.

Rotating the sample within the equatorial plane of the open split resonator induced modifications in $f_{res}$, as shown in Figure 15 (experimental $S_{21}$) for a 1 mm split gap. As the sample rotates, two resonant peaks, referred to as peak 1 and peak 2, appear based on the alignment of the material’s effective permittivity tensor with the TE_111_ mode’s electric field. A single resonance peak is observed when the rotation aligns the tensor with the principal axes of $\varepsilon_{eff}$, diagonalizing it. The rotation angle that produces peak 1 alone is defined as 0°. At the rotation angle of 0°, the anisotropy tensor components in the sample’s plane are parallel $\varepsilon_{eff,||}$ and perpendicular $\varepsilon_{eff,⊥}$ to the director direction of the electric field $\vec{E}$, while $\varepsilon_{eff,z}$ is orthogonal to the plane. Further rotation induces mode degeneration, causing the emergence of peak 2 alongside peak 1. Extending the rotation to 90° vanishes peak 1, leaving only peak 2.

Numerical calculations were used to identify the effective permittivity that reproduces $S_{21}$ results at 0°. Then, the orientation tensor that produced that effective permittivity was determined by using the interconnected straight filament approach, with permittivities set to 9.95−j0.0298 for alumina and 1−j0.0006 for air. The resulting orientation tensor at 0° is T = $\left[0.349\;0\;0;0\;0.328\;0;0\;0\;0.323\right]$. Further, simulations of the resonator were then performed by performing in-plane rotations of tensor T at the angles of 0°, 45°, and 90°, with the resulting simulated $S_{21}$ shown in Figure 15. While minor differences are observed between the experimental and simulated $S_{21}$—likely attributable to variations in fiber orientation introduced during the imperfect manual rotation of the fabric—another contributing factor is the use of averaged sample characteristics like thickness and porosity (Table 7). Nevertheless, there is a good agreement between these simulated and actual measured $S_{21}$ plots that confirm the applicability of this approach.

## 4. Conclusions

This work investigated the efficacy of mixing relations for estimating the anisotropic effective permittivity of fibrous media, focusing on characterizing inclusion geometries as sinusoidal weaves and interconnected elements to mimic the curvature of fibers. This included employing straight filaments and ellipsoids based on models such as the Maxwell–Garnett and Traditional Anisotropic Bruggeman. The effectiveness of these approaches was compared for both dispersed fibers and woven fabrics through numerical calculations.

The investigation revealed that traditional modeling with ellipsoids, while offering preliminary insights, does not adequately represent the complex geometry and inherent anisotropy of fibrous media, especially in woven fabrics. The application of an interconnected straight filament approach demonstrated enhanced capability in accurately modeling these characteristics, leading to more precise estimations of effective permittivity across various orientations and conditions. The findings highlight the importance of geometric fidelity in modeling real-world materials. Although the sinusoidal wave approach yields good estimates, its application is confined to materials with sinusoidal-shaped inclusions, a limited range of porosities from 0.8 to 1, and fibers in the micrometer scale. Conversely, the interconnected approach offers broader versatility, extending the scope of accurately modeled materials to include any curved shapes and porosity, with its applicability only constrained by the models’ alignment with effective medium theory.

In conclusion, this study provides a comprehensive framework for estimating the effective permittivity of anisotropic fibrous media, representing a significant advancement over conventional models. These novel approaches offer the potential to optimize material orientation arrangements in fabric antenna design, enhancing signal transmission efficiency. Furthermore, it could improve the performance of electromagnetic shielding textiles, ensuring better control of signal attenuation in sensitive environments. Additionally, these approaches may facilitate the design of more efficient dielectric layers in high-frequency fiber-reinforced circuits. Overall, the approaches provide benefits in the design and optimization of fibrous materials for engineering applications.

## Figures and Tables

**Table 1:** Matrix ab component values of $C_{\left(\theta_{f},ab\right)},F_{\left(\theta_{f},ab\right)},J_{\left(\theta_{f},ab\right)},N_{\left(\theta_{f},ab\right)},B_{ab},E_{ab},M_{ab},$ and $P_{ab}$.

Coefficient	Matrix *ij*−Component of Coefficients								
	11	12	13	21	22	23	31	32	33
$C_{0,ab}$	0.1624	−0.4050	−0.4050	−0.4050	1.0100	0.0000	−0.4050	0.0000	1.0100
$C_{90,ab}$	0.1624	0.3530	0.3530	0.3530	0.1695	0.0000	0.3035	0.0000	0.1695
$F_{0,ab}$	−0.0810	−0.0280	−0.0280	−0.0280	0.0242	0.0000	−0.0280	0.0000	0.0242
$F_{90,ab}$	−0.0810	0.0040	0.0040	0.0040	−0.0790	0.0000	0.0040	0.0000	−0.0790
$J_{0,ab}$	−0.8025	−0.3466	−0.3466	−0.3466	0.8318	0.0001	−0.3466	0.0001	0.8318
$J_{90,ab}$	−0.8025	0.3466	0.3466	0.3466	0.0001	0.0001	0.3466	0.0001	0.0001
$N_{0,ab}$	−0.1210	0.0001	0.0001	0.0001	0.1055	0.0001	0.0001	0.0001	0.1055
$N_{90,ab}$	−0.1210	0.0750	0.0750	0.0750	0.0001	0.0001	0.0750	0.0001	0.0001
$B_{ab}$	−0.2444	−0.1440	−0.1440	−0.1440	−0.1900	0.0000	−0.1440	0.0000	−0.1900
$E_{ab}$	−0.2685	−0.5500	−0.5500	−0.5500	−0.1780	0.0000	−0.5500	0.0000	−0.1780
$M_{ab}$	0.9650	−0.0530	−0.0530	−0.0530	0.1695	0.0000	−0.0530	0.0000	0.1695
$P_{ab}$	0.0420	−0.0280	−0.0280	−0.0280	−0.0790	0.0000	−0.0280	0.0000	−0.0790

**Table 2:** *κ*_ab_ values describing straight filaments.

*κ*_ab_	Value	*κ*_ab_	Value
$\kappa_{11}$	$\frac{1}{\delta_{i}\delta_{m}\varepsilon_{i}}\left(\frac{2\delta_{m}}{\left(1+{\delta_{m}}^{2}\right)}\left(\varepsilon_{i}-\varepsilon_{m}\right)+\left(\varepsilon_{w}^{+}-\varepsilon_{i}\right)\right)$	$\kappa_{12}=\kappa_{21}$	−0.0560+j0.0300
$\kappa_{22}$	$\frac{1}{\delta_{i}\delta_{m}\varepsilon_{i}}\left(\frac{2\delta_{m}}{\left(1+{\delta_{m}}^{2}\right)}\left(\varepsilon_{i}-\varepsilon_{m}\right)+\left(\varepsilon_{w}^{-}-\varepsilon_{i}\right)+\left(\varepsilon_{HS}^{-}-\varepsilon_{w}^{-}\right)\left(0.8865-j0.0224\right)\right)$	$\kappa_{13}=\kappa_{31}$	−0.0560+j0.0300
$k_{33}$	$\frac{1}{\delta_{i}\delta_{m}\varepsilon_{i}}\left(\frac{2\delta_{m}}{\left(1+{\delta_{m}}^{2}\right)}\left(\varepsilon_{i}-\varepsilon_{m}\right)+\left(\varepsilon_{w}^{-}-\varepsilon_{i}\right)+\left(\varepsilon_{HS}^{-}-\varepsilon_{w}^{-}\right)\left(0.8865-j0.0224\right)\right)$	$k_{23}=\kappa_{32}$	0.0000−j0.0000

**Table 3:** DF-13-4500 fiber fabric properties (superscripts: ^1^ according to 3M company brochure, ^2^ Pritzkow et al. [21]; and ^3^ calculated using the density of Al_2_O_3_ = 3.9 g·cm^−3^).

Fabric	Warp/Fill Thread ^1^ Count/cm^−1^	Thickness ^2^/mm	Areal Density ^2^/g·m^−2^	Porosity ^3^/-
DF-13-4500	4.7/4.7	0.518	500	0.753

**Table 4:** Properties of the evaluated dispersed fibrous models.

Model	*δ*_m_	*T* (All Fibers)	Fiber-Types (fN) Composing Model					
			fN	$\delta_{i,fN}$	$L_{f,fN}$	$H_{f,fN}$	$\theta_{f,fN}$	$T_{dir,fN}$
1	0.9018	$\left[\begin{array}{ccc} 1 & 0 & 0\\ 0 & 0 & 0\\ 0 & 0 & 0 \end{array}\right]$	1	0.0982	∞	0	0°	$\left[\begin{array}{ccc} 1 & 0 & 0\\ 0 & 0 & 0\\ 0 & 0 & 0 \end{array}\right]$
2	0.9018	$\left[\begin{array}{ccc} 0 & 0 & 0\\ 0 & 1 & 0\\ 0 & 0 & 0 \end{array}\right]$	1	0.0982	∞	0	0°	$\left[\begin{array}{ccc} 0 & 0 & 0\\ 0 & 1 & 0\\ 0 & 0 & 0 \end{array}\right]$
3	0.9194	$\left[\begin{array}{ccc} 0.5 & 0.5 & 0\\ 0.5 & 0.5 & 0\\ 0 & 0 & 0 \end{array}\right]$	1	0.0806	∞	0	0°	$\left[\begin{array}{ccc} 0.5 & 0.5 & 0\\ 0.5 & 0.5 & 0\\ 0 & 0 & 0 \end{array}\right]$
4	0.9026	$\left[\begin{array}{ccc} 0.5721 & 0.0004 & -0.0015\\ 0.0004 & 0.0085 & -0.0060\\ -0.0015 & -0.0060 & 0.4194 \end{array}\right]$	1	0.0974	200	40	0°	$\left[\begin{array}{ccc} 1 & 0 & 0\\ 0 & 0 & 0\\ 0 & 0 & 0 \end{array}\right]$
5	0.9026	$\left[\begin{array}{ccc} 0.0086 & -0.0016 & -0.0093\\ -0.0016 & 0.6352 & -0.0098\\ -0.0093 & -0.0098 & 0.3562 \end{array}\right]$	1	0.0974	200	40	90°	$\left[\begin{array}{ccc} 0 & 0 & 0\\ 0 & 1 & 0\\ 0 & 0 & 0 \end{array}\right]$
6	0.9026	$\left[\begin{array}{ccc} 0.3568 & -0.0072 & 0.0007\\ -0.0072 & 0.6355 & 0.0070\\ 0.0007 & 0.0070 & 0.0077 \end{array}\right]$	1	0.0974	200	40	0°	$\left[\begin{array}{ccc} 0 & 0 & 0\\ 0 & 1 & 0\\ 0 & 0 & 0 \end{array}\right]$
7	0.9061	$\left[\begin{array}{ccc} 0.3184 & 0.2986 & 0.0092\\ 0.2986 & 0.3208 & -0.0082\\ 0.0092 & -0.0082 & 0.3608 \end{array}\right]$	1	0.0939	200	40	0°	$\left[\begin{array}{ccc} 0.5 & 0.5 & 0\\ 0.5 & 0.5 & 0\\ 0 & 0 & 0 \end{array}\right]$
8	0.9061	$\left[\begin{array}{ccc} 0.3617 & 0.0104 & 0.0020\\ 0.0104 & 0.3269 & 0.2996\\ 0.0020 & 0.2996 & 0.3114 \end{array}\right]$	1	0.0939	200	40	0°	$\left[\begin{array}{ccc} 0 & 0 & 0\\ 0 & 0.5 & 0.5\\ 0 & 0.5 & 0.5 \end{array}\right]$
9	0.9061	$\left[\begin{array}{ccc} 0.3247 & -0.0120 & 0.3002\\ -0.0120 & 0.3657 & 0.0003\\ 0.3002 & 0.0003 & 0.3096 \end{array}\right]$	1	0.0939	200	40	0°	$\left[\begin{array}{ccc} 0.5 & 0 & 0.5\\ 0 & 0 & 0\\ 0.5 & 0 & 0.5 \end{array}\right]$
10	0.9021	$\left[\begin{array}{ccc} 0.1164 & 0.1137 & 0.0050\\ 0.1137 & 0.1163 & 0.0048\\ 0.0050 & 0.0048 & 0.7674 \end{array}\right]$	1	0.0610	∞	0	0°	$\left[\begin{array}{ccc} 0 & 0 & 0\\ 0 & 0 & 0\\ 0 & 0 & 1 \end{array}\right]$
			2	0.0369	200	40	90°	$\left[\begin{array}{ccc} 0.5 & 0.5 & 0\\ 0.5 & 0.5 & 0\\ 0 & 0 & 0 \end{array}\right]$
11	0.9501	$\left[\begin{array}{ccc} 0.5069 & 0.0430 & 0.0121\\ 0.0430 & 0.2880 & -0.0001\\ 0.0121 & -0.0001 & 0.2050 \end{array}\right]$	1	0.0225	200	40	90°	$\left[\begin{array}{ccc} 0.5 & 0.5 & 0\\ 0.5 & 0.5 & 0\\ 0 & 0 & 0 \end{array}\right]$
			2	0.0274	300	50	0°	$\left[\begin{array}{ccc} 1 & 0 & 0\\ 0 & 0 & 0\\ 0 & 0 & 0 \end{array}\right]$
12	0.9497	$\left[\begin{array}{ccc} 0.2378 & 0.0000 & 0.0149\\ 0.0000 & 0.4216 & 0.0149\\ 0.0149 & 0.0149 & 0.3405 \end{array}\right]$	1	0.0163	75	15	0°	$\left[\begin{array}{ccc} 0 & 0 & 0\\ 0 & 0 & 0\\ 0 & 0 & 1 \end{array}\right]$
			2	0.0206	100	10	0°	$\left[\begin{array}{ccc} 0 & 0 & 0\\ 0 & 1 & 0\\ 0 & 0 & 0 \end{array}\right]$
			3	0.0134	200	20	45°	$\left[\begin{array}{ccc} 0.10 & 0.00 & 0.05\\ 0.00 & 0.10 & 0.05\\ 0.05 & 0.05 & 0.80 \end{array}\right]$

**Table 5:** Estimated *ε*_eff,xx_ using wave-curved and interconnected elements approaches.

Model		Simulation	Mixing Relations			
			Sinusoidal Wave-Curved	Interconnected Straight Fibers	Maxwell–Garnett	Bruggeman
		*ε*_eff,xx_	*ε*_eff,xx_	*ε*_eff,xx_	*ε*_eff,xx_	*ε*_eff,xx_
Straight fibers	1	$\left(1.884\pm 0.000\right)-j\left(0.491\pm 0.000\right)$	1.884−j0.491	1.884−j0.491	1.884−j0.491	1.884−j0.491
	2	$\left(1.233\pm 0.000\right)-j\left(0.032\pm 0.000\right)$	1.233−j0.032	1.233−j0.031	1.182−j0.016	1.196−j0.020
	3	$\left(1.431\pm 0.002\right)-j\left(0.171\pm 0.006\right)$	1.430−j0.174	1.427−j0.171	1.436−j0.208	1.441−j0.209
Sinusoidal fibers	4	$\left(1.598\pm 0.000\right)-j\left(0.283\pm 0.001\right)$	1.599−j0.283	1.600−j0.292	1.593−j0.297	1.595−j0.297
	5	$\left(1.231\pm 0.000\right)-j\left(0.035\pm 0.001\right)$	1.231−j0.031	1.236−j0.034	1.190−j0.022	1.197−j0.024
	6	$\left(1.461\pm 0.000\right)-j\left(0.178\pm 0.001\right)$	1.460−j0.179	1.461−j0.193	1.454−j0.204	1.460−j0.207
	7	$\left(1.405\pm 0.001\right)-j\left(0.144\pm 0.001\right)$	1.407−j0.141	1.399−j0.139	1.396−j0.166	1.400−j0.167
	8	$\left(1.444\pm 0.000\right)-j\left(0.173\pm 0.001\right)$	1.443−j0.173	1.447−j0.188	1.440−j0.198	1.445−j0.201
	9	$\left(1.406\pm 0.000\right)-j\left(0.147\pm 0.001\right)$	1.407−j0.141	1.403−j0.142	1.400−j0.169	1.404−j0.170
Multiple fibers	10	$\left(1.298\pm 0.000\right)-j\left(0.078\pm 0.000\right)$	1.297−j0.083	1.299−j0.072	1.274−j0.080	1.283−j0.083
	11	$\left(1.278\pm 0.000\right)-j\left(0.131\pm 0.001\right)$	1.277−j0.128	1.282−j0.131	1.275−j0.133	1.276−j0.133
	12	$\left(1.198\pm 0.000\right)-j\left(0.069\pm 0.001\right)$	1.201−j0.066	1.195−j0.070	1.182−j0.070	1.183−j0.070
RMSE		Models 1–3 (straight fibers)	0.001+j0.002	0.002+j0.001	0.023−j0.014	0.016−j0.017
		Models 4–9 (sinusoidal fibers)	0.001+j0.003	0.002−j0.009	0.002+j0.011	0.001+j0.018
		Models 10–12 (multiple fibers)	0.001+j0.003	0.001+j0.002	0.017+j0.001	0.012−j0.003
Total RMSE		Models 1–12	0.001+j0.003	0.001−j0.007	0.011−j0.006	0.006−j0.013
Average RMSE (straight, sinusoidal and multiple fibers)			0.001+j0.003	0.002−j0.002	0.014−j0.001	0.010−j0.011

**Table 6:** Properties of the evaluated plain-woven fabrics.

Model	*δ*_m_/-	*T*/-	*P*/µm
1	0.461	$\left[\begin{array}{ccc} 0.3519 & 0 & 0\\ 0 & 0.3519 & 0\\ 0 & 0 & 0.2962 \end{array}\right]$	500
2	0.505	$\left[\begin{array}{ccc} 0.3540 & 0 & 0\\ 0 & 0.3540 & 0\\ 0 & 0 & 0.2920 \end{array}\right]$	535
3	0.530	$\left[\begin{array}{ccc} 0.3566 & 0 & 0\\ 0 & 0.3566 & 0\\ 0 & 0 & 0.2868 \end{array}\right]$	556
4	0.559	$\left[\begin{array}{ccc} 0.3615 & 0 & 0\\ 0 & 0.3615 & 0\\ 0 & 0 & 0.2770 \end{array}\right]$	588
5	0.625	$\left[\begin{array}{ccc} 0.3736 & 0 & 0\\ 0 & 0.3736 & 0\\ 0 & 0 & 0.2528 \end{array}\right]$	666
6	0.698	$\left[\begin{array}{ccc} 0.3915 & 0 & 0\\ 0 & 0.3915 & 0\\ 0 & 0 & 0.2170 \end{array}\right]$	800
7	0.764	$\left[\begin{array}{ccc} 0.4114 & 0 & 0\\ 0 & 0.4114 & 0\\ 0 & 0 & 0.1772 \end{array}\right]$	1000

**Table 7:** Sample characteristics after yarn modifications and drying (superscript: ^1^ calculated using areal density from Table 3).

Maximum Weft Yarn Height/mm	Maximum Warp Yarn Height/mm	Average Thickness/mm	Porosity ^1^/-
0.501	0.530	0.440 ± 0.92	0.7086 ± 0.05

## Data Availability

Data are contained within the article.

## Appendix A

### Appendix A.1. ε_eff,x_ Calculation Examples

The section below demonstrates how to calculate $\varepsilon_{eff,xx}$ reported in Table 5 for models listed in Table 4. Specifically, we detail the calculations for models 3, 7, and 10. Models 3 and 7 consist of a single fiber type $\left(fN=1\right)$ of straight and sinusoidal fibers, respectively. In contrast, model 10 features two fiber types: the first $\left(fN=1\right)$ is straight and the second $\left(fN=2\right)$ is sinusoidal, with each fN possessing a distinct fiber orientation.

### Appendix A.2. Calculations Using the Sinusoidal Wave-Curved Approach

Applying the sinusoidal wave-curved approach requires knowing the fiber’s parameters, $d_{f,fN},H_{f,fN},L_{f,fN},$ and $\theta_{f,fN}$, as well as the fiber’s direction, indicated by $T_{dir}$ for each group of fibers fN comprising a fibrous model. For the calculated models, these parameters are reported in Table 4.

1.1 The first step involves calculating the coefficients $C_{\left(\theta_{f},ab\right)},F_{\left(\theta_{f},ab\right)},J_{\left(\theta_{f},ab\right)},\;andN_{\left(\theta_{f},ab\right)}$ at their respective $\theta_{f}$ by using $S=\left(1+\cos\left(2\theta_{f}\right)\right)/2$ and $\Psi_{\left(\theta_{f},ab\right)}=\left(\Psi_{\left(0{}^{\circ},ab\right)}-\Psi_{\left(90{}^{\circ},ab\right)}\right)S+\Psi_{\left(90{}^{\circ},ab\right)}$, and considering matrix components from Table 1.
  For fN=1 of models 3, 7, and 10, $\theta_{f}=0{}^{\circ},$ leading to S=1, for which the coefficient results are	
  $C_{0{}^{\circ}}=\left[\begin{array}{ccc} \;\;\;0.1624 & -0.4050 & -0.4050\\ -0.4050 & \;\;\;1.0100 & \;\;\;0.0000\\ -0.4050 & \;\;\;0.0000 & \;\;\;1.0100 \end{array}\right]$	$J_{0{}^{\circ}}=\left[\begin{array}{ccc} -0.8025 & -0.3466 & -0.3466\\ -0.3466 & \;\;\;0.8318 & \;\;\;0.0001\\ -0.3466 & \;\;\;0.0001 & \;\;\;0.8318 \end{array}\right]$
  $F_{0{}^{\circ}}=\left[\begin{array}{ccc} -0.0810 & -0.0280 & -0.0280\\ -0.0280 & \;\;\;0.0242 & \;\;\;0.0000\\ -0.0280 & \;\;\;0.0000 & \;\;\;0.0242 \end{array}\right]$	$N_{0{}^{\circ}}=\left[\begin{array}{ccc} -0.1210 & 0.0001 & 0.0001\\ \;\;\;0.0001 & 0.1055 & 0.0001\\ \;\;\;0.0001 & 0.0001 & 0.1055 \end{array}\right]$
1.2 Using the determined C, F, J, and N, as well as B, E, M, and P (from Table 1), K (containing $\kappa_{ab}$) is calculated from Equation (14) as
  Model 3: $K=\left[\begin{array}{ccc} \;\;\;0.9650-j0.0420 & -0.0530+j0.0280 & -0.0530+j0.0280\\ -0.0530+j0.0280 & \;\;\;0.1695+j0.0790 & \;\;\;0.0000+j0.0000\\ -0.0530+j0.0280 & \;\;\;0.0000+j0.0000 & \;\;\;0.1695+j0.0790 \end{array}\right]$
  Model 7: $K=\left[\begin{array}{ccc} \;\;\;0.6183+j0.0172 & -0.1351+j0.0280 & -0.1351+j0.0280\\ -0.1351+j0.0280 & \;\;\;0.4457+j0.0484 & \;\;\;0.0000+j0.0000\\ -0.1351+j0.0280 & \;\;\;0.0000+j0.0000 & \;\;\;0.4457+j0.0484 \end{array}\right]$
  Model 10: $K=\left[\begin{array}{ccc} \;\;\;0.9650-j0.0420 & -0.0530+j0.0280 & -0.0530+j0.0280\\ -0.0530+j0.0280 & \;\;\;0.1695+j0.0790 & \;\;\;0.0000+j0.0000\\ -0.0530+j0.0280 & \;\;\;0.0000+j0.0000 & \;\;\;0.1695+j0.0790 \end{array}\right]$
1.3 Then, $\kappa_{ab}$- and $t_{ab}$-components from K and $T_{dir,fN}$ are used alongside $\delta_{m,fN},\;\delta_{i,fN},\;\varepsilon_{m}$, and $\varepsilon_{i}$ in Equation (10) (using $T_{dir,fN}$ as T) to obtain $\varepsilon_{eff,xx}$. Note that in the case of calculating $\varepsilon_{eff,yy}$ and $\varepsilon_{eff,zz}$, $T_{dir}$ must be tensor rotated to the z- and *y*-axis, respectively.
  Model 3	Model 7	Model 10
  $\varepsilon_{eff,xx}=1.8838-j0.4911$	$\varepsilon_{eff,xx}=1.4071-j0.1413$	$\varepsilon_{eff,xx}=1.2747-j0.0263$

The calculated $\varepsilon_{eff,xx}$ corresponds to that of the first fiber type fN=1 of models 3, 7, and 10. However, since Model 10 includes a second fiber type fN=2, the calculation procedure must be reiterated for this additional fiber type. The procedure is identical, except that the permittivity of the continuous medium (or host phase) is now represented by the previously determined effective permittivity, $\varepsilon_{m}=\varepsilon_{eff,xx}.$ This adjustment accounts for the addition of the second fiber type into the already calculated effective medium (which consists of the first fiber type and the host medium).

For the second fiber type fN=2 of model 10, $\theta_{f}=90{}^{\circ},$ which gives S=0 and	
$C_{90{}^{\circ}}=\left[\begin{array}{ccc} 0.1624 & 0.3530 & 0.3530\\ 0.3530 & 0.1695 & 0.0000\\ 0.3530 & 0.0000 & 0.1695 \end{array}\right]$	$J_{90{}^{\circ}}=\left[\begin{array}{ccc} -0.8025 & -0.3466 & -0.3466\\ -0.3466 & \;\;\;0.0001 & \;\;\;0.0001\\ -0.3466 & \;\;\;0.0001 & \;\;\;0.0001 \end{array}\right]$
$F_{90{}^{\circ}}=\left[\begin{array}{ccc} -0.0810 & \;\;\;0.0400 & \;\;\;0.0400\\ \;\;\;0.0400 & -0.0790 & \;\;\;0.0000\\ \;\;\;0.0400 & \;\;\;0.0000 & -0.0790 \end{array}\right]$	$N_{90{}^{\circ}}=\left[\begin{array}{ccc} -0.1210 & 0.0750 & 0.0750\\ \;\;\;0.0750 & 0.0001 & 0.0001\\ \;\;\;0.0750 & 0.0001 & 0.0001 \end{array}\right]$

Proceed with calculating K for fN=2,as follows:

Model 10: $K=\left[\begin{array}{ccc} 0.6188+j0.0171 & 0.0526-j0.0231 & 0.0526-j0.0231\\ 0.0526-j0.0231 & 0.1695+j0.0790 & 0.0000+j0.0000\\ 0.0526-j0.0231 & 0.0000+j0.0000 & 0.1695+j0.0790 \end{array}\right]$

Finally, the $\varepsilon_{eff,xx}$ for the multiple fibres of model 10 is

Model 10: $\varepsilon_{eff,xx}=1.2966-j0.0827$

### Appendix A.3. Calculations Using the Interconnected Straight Fibers Approach

Contrary to the sinusoidal wave-curved approach, which utilizes $T_{dir}$, the interconnected elements approach employs the tensor $T_{\Lambda }$, as this tensor sufficiently describes all fibers within a fibrous medium. As detailed in Section 2.5, one method to determine this tensor is through the use of the SLD algorithm [22]. The following points summarize the calculation sequence for $\varepsilon_{eff,xx}$.

1.1 Calculate $\sum_{a=1}^{3}\sum_{b=1}^{3}\left|t_{\Lambda ,ab}\right|\kappa_{ab}\;$ by using $T_{\Lambda }$ (see Table 4) and the geometrical tensor K given for a geometry corresponding to straight filaments (from Table 2).
  Model 3: $\sum_{a=1}^{3}\sum_{b=1}^{3}\left|t_{\Lambda ,ab}\right|\kappa_{ab}\;=0.9649-j0.0420$
  Model 7: $\sum_{a=1}^{3}\sum_{b=1}^{3}\left|t_{\Lambda ,ab}\right|\kappa_{ab}\;=0.3883+j0.0589$
  Model 10: $\sum_{a=1}^{3}\sum_{b=1}^{3}\left|t_{\Lambda ,ab}\right|\kappa_{ab}\;=0.5601+j0.0182$
1.2 Calculate $\varepsilon_{eff,xx}$ by using Equation (10).
  Model 3	Model 7	Model 10
  $\varepsilon_{eff,xx}=1.8838-j0.4911$	$\varepsilon_{eff,xx}=1.399-j0.139$	$\varepsilon_{eff,xx}=1.2816-j0.1307$

### Appendix A.4. Calculations Using the Interconnected Elongated Ellipsoids from the Maxwell–Garnett and Traditional Anisotropic Bruggeman Mixing Relations

1.1 Calculate the eigenvalues $T_{D}$ and eigenvectors V of the orientation tensor T.
  Model 3	$V=\left[\begin{array}{ccc} -0.7071 & 0.0000 & 0.7071\\ \;\;\;0.7071 & 0.0000 & 0.7071\\ \;\;\;0.0000 & 1.0000 & 0.0000 \end{array}\right]$	$T_{D}=\left[\begin{array}{ccc} 0.0000 & 0.0000 & 0.0000\\ 0.0000 & 0.0000 & 0.0000\\ 0.0000 & 0.0000 & 1.0000 \end{array}\right]$
  Model 7	$V=\left[\begin{array}{ccc} -0.7081 & \;\;\;0.0237 & -0.7057\\ \;\;\;0.7052 & -0.0275 & -0.7085\\ \;\;\;0.0362 & \;\;\;0.9993 & -0.0028 \end{array}\right]$	$T_{D}=\left[\begin{array}{ccc} 0.0205 & 0.0000 & 0.0000\\ 0.0000 & 0.3613 & 0.0000\\ 0.0000 & 0.0000 & 0.6182 \end{array}\right]$
  Model 10	$V=\left[\begin{array}{ccc} \;\;\;0.7069 & \;\;\;0.7072 & 0.0092\\ -0.7073 & \;\;\;0.7068 & 0.0090\\ -0.0001 & -0.0129 & 0.9999 \end{array}\right]$	$T_{D}=\left[\begin{array}{ccc} 0.0027 & 0.0000 & 0.0000\\ 0.0000 & 0.2299 & 0.0000\\ 0.0000 & 0.0000 & 0.7674 \end{array}\right]$
1.2 An effective permittivity for each diagonal component ($\varepsilon_{eff,diag,1,1}$, $\varepsilon_{eff,diag,2,2},$ and $\varepsilon_{eff,diag,3,3}$) is calculated by considering the volume fraction $\delta_{i\left(n\right)}$ of each diagonal component and host medium $\delta_{m\left(n\right)}=1-\delta_{i\left(n\right)}$. $\delta_{i,\left(n\right)}$ is determined through an iterative process, using the formulas
  $$\delta_{m1}=\frac{1-\left(1-\delta_{m}\right)T_{D,\left(1,1\right)}}{1}\;$$
  $$\delta_{m2}=\frac{1-\left(1-\delta_{m}\right)\left(T_{D,\left(1,1\right)}+T_{D,\left(2,2\right)}\right)}{1-T_{D,\left(1,1\right)}}$$
  $$\delta_{m3}=\frac{1-\left(1-\delta_{m}\right)\left(T_{D,\left(1,1\right)}+T_{D,\left(2,2\right)}+T_{D,\left(3,3\right)}\right)}{1-T_{D,\left(1,1\right)}-T_{D,\left(2,2\right)}}$$

Accordingly, $\delta_{m\left(n\right)}$ of the considered models gives

Model 3:	$\delta_{m1}=1.0000$	$\delta_{m2}=1.0000$	$\delta_{m3}=0.9194$
Model 7:	$\delta_{m1}=0.9981$	$\delta_{m2}=0.9660$	$\delta_{m3}=0.9398$
Model 10:	$\delta_{m1}=0.9997$	$\delta_{m2}=0.9775$	$\delta_{m3}=0.9231$

1.3 The MG and TAB mixing relations are used in an iterative way, as follows:
  MG mixing relation
  $\varepsilon_{m2}=\varepsilon_{m1}\left(1+\delta_{i1}\frac{\varepsilon_{i}-\varepsilon_{m1}}{\varepsilon_{m1}+\delta_{m1}\left[\begin{array}{ccc} 0 & 0 & 0\\ 0 & 0.5 & 0\\ 0 & 0 & 0.5 \end{array}\right]\left(\varepsilon_{i}-\varepsilon_{m1}\right)}\right)$
  $\varepsilon_{m3}=\varepsilon_{m2}\left(1+\delta_{i2}\frac{\varepsilon_{i}-\varepsilon_{m2}}{\varepsilon_{m2}+\delta_{m2}\left[\begin{array}{ccc} 0.5 & 0 & 0\\ 0 & 0 & 0\\ 0 & 0 & 0.5 \end{array}\right]\left(\varepsilon_{i}-\varepsilon_{m2}\right)}\right)$
  $\varepsilon_{eff}=\varepsilon_{m3}\left(1+\delta_{i3}\frac{\varepsilon_{i}-\varepsilon_{m3}}{\varepsilon_{m3}+\delta_{m3}\left[\begin{array}{ccc} 0.5 & 0 & 0\\ 0 & 0.5 & 0\\ 0 & 0 & 0 \end{array}\right]\left(\varepsilon_{i}-\varepsilon_{m3}\right)}\right)$
  TAB mixing relation
  $\delta_{m1}\frac{\varepsilon_{m1}-\varepsilon_{m2}}{\varepsilon_{m2}+\left[\begin{array}{ccc} 0 & 0 & 0\\ 0 & 0.5 & 0\\ 0 & 0 & 0.5 \end{array}\right]\left(\varepsilon_{m}-\varepsilon_{m2}\right)}+\delta_{i1}\frac{\varepsilon_{i}-\varepsilon_{m2}}{\varepsilon_{m2}+\left[\begin{array}{ccc} 0 & 0 & 0\\ 0 & 0.5 & 0\\ 0 & 0 & 0.5 \end{array}\right]\left(\varepsilon_{i}-\varepsilon_{m2}\right)}$
  $\delta_{m2}\frac{\varepsilon_{m2}-\varepsilon_{m3}}{\varepsilon_{m3}+\left[\begin{array}{ccc} 0.5 & 0 & 0\\ 0 & 0 & 0\\ 0 & 0 & 0.5 \end{array}\right]\left(\varepsilon_{m2}-\varepsilon_{m3}\right)}+\delta_{i2}\frac{\varepsilon_{i}-\varepsilon_{m3}}{\varepsilon_{m3}+\left[\begin{array}{ccc} 0.5 & 0 & 0\\ 0 & 0 & 0\\ 0 & 0 & 0.5 \end{array}\right]\left(\varepsilon_{i}-\varepsilon_{m3}\right)}$
  $\delta_{m3}\frac{\varepsilon_{m3}-\varepsilon_{eff}}{\varepsilon_{eff}+\left[\begin{array}{ccc} 0.5 & 0 & 0\\ 0 & 0.5 & 0\\ 0 & 0 & 0 \end{array}\right]\left(\varepsilon_{m3}-\varepsilon_{eff}\right)}+\delta_{i}\frac{\varepsilon_{i}-\varepsilon_{eff}}{\varepsilon_{eff}+\left[\begin{array}{ccc} 0.5 & 0 & 0\\ 0 & 0.5 & 0\\ 0 & 0 & 0 \end{array}\right]\left(\varepsilon_{i}-\varepsilon_{eff}\right)}$
  where
  $\varepsilon_{m1}=\left[\begin{array}{ccc} \varepsilon_{m,\left(1,1\right)} & 0 & 0\\ 0 & \varepsilon_{m,\left(1,1\right)} & 0\\ 0 & 0 & \varepsilon_{m,\left(1,1\right)} \end{array}\right],$ $\varepsilon_{m2}=\left[\begin{array}{ccc} \varepsilon_{m2,\left(1,1\right)} & 0 & 0\\ 0 & \varepsilon_{m2,\left(2,2\right)} & 0\\ 0 & 0 & \varepsilon_{m2,\left(3,3\right)} \end{array}\right]$,
  $\varepsilon_{m3}=\left[\begin{array}{ccc} \varepsilon_{m3,\left(1,1\right)} & 0 & 0\\ 0 & \varepsilon_{m3,\left(2,2\right)} & 0\\ 0 & 0 & \varepsilon_{m3,\left(3,3\right)} \end{array}\right],$ and $\varepsilon_{i}=\left[\begin{array}{ccc} \varepsilon_{i,\left(1,1\right)} & 0 & 0\\ 0 & \varepsilon_{i,\left(1,1\right)} & 0\\ 0 & 0 & \varepsilon_{i,\left(1,1\right)} \end{array}\right]$.

Finally, giving $\varepsilon_{eff,xx}$

	**MG**	**TAB**
Model 3:	$\varepsilon_{eff,xx}=1.436-j0.208$	$\varepsilon_{eff,xx}=1.441-j0.209$
Model 7:	$\varepsilon_{eff,xx}=1.396-j0.166$	$\varepsilon_{eff,xx}=1.400-j0.167$
Model 10:	$\varepsilon_{eff,xx}=1.274-j0.080$	$\varepsilon_{eff,xx}=1.283-j0.083$
